# Dairy Bioactive Compounds as Precision Modulators of Gut Microbiota: From Molecular Mechanisms to Personalized Immunometabolic Health

**DOI:** 10.3390/foods15112024

**Published:** 2026-06-04

**Authors:** Omar A. Alhaj, Nour A. Elsahoryi, Haitham A. Jahrami

**Affiliations:** 1 Department of Nutrition, Faculty of Pharmacy and Medical Sciences, University of Petra, Amman 11196, Jordan; nour.elsahoryi@uop.edu.jo; 2 Government Hospitals, Manama 329, Bahrain; hjahrami@hospitals.gov.bh; 3 Department of Psychiatry, College of Medicine and Medical Sciences, Arabian Gulf University, Manama 329, Bahrain

**Keywords:** dairy bioactivity, gut microbiota, precision nutrition, immunometabolism, whey proteins, milk oligosaccharides, intestinal barrier, personalized health

## Abstract

The gut microbiota (GM) has become a key mediator of host health, with dietary manipulations promising ways of modulating the microbiome. This review focuses on the role of dairy bioactive (DB) compounds as precision modulators of intestinal microecology, including the whey proteins (WPs), including lactoferrin (LF), α-lactalbumin (LA), β-lactoglobulin, lysozyme (LZ), lactoperoxidase, glycomacropeptide (GMP), milk oligosaccharides (MOs), and bioactive peptides (BPs). This review compiles the existing evidence illustrating their dual-action mechanism through direct prebiotic activity and the promotion of beneficial taxa (*Bifidobacterium*, *Lactobacillus*, *Faecalibacterium*), along with selective antimicrobial activity and pathogen suppression. These compounds improve intestinal barrier integrity through tight junction (TJ) protein regulation, regulating short-chain fatty acid production, and modulating immune signaling pathways. Clinical evidence shows significant benefits in metabolism and inflammation among various populations. However, individual responses vary according to host factors such as enterotypes, FUT2 genotype, and baseline microbiota composition, suggesting the need for personalized intervention strategies. This review addresses critical knowledge gaps in dose–response relationships, long-term efficacy, and mechanistic pathways and suggests future directions for precision nutrition. By modifying molecular mechanisms in clinical applications, we have identified DB compounds as promising candidates for targeted modulation of the microbiota to optimize health and disease management. The review also brings together molecular mechanistic and clinically implementable, personalized dietary strategies, which have not been fully captured by previous reviews. It pinpoints gaps in knowledge related to dose–response characterization, long-term trial design, and multi-omics stratification that collectively define a new precision nutrition framework. In this approach, dairy-based intervention is planned for each person based on their microbial, genetic, and metabolic characteristics.

## 1. Introduction

### 1.1. Gut Microbiota and Paradigm of Health

The GM is the complex community of Archaea, Bacteria, Eukarya, and viruses that live in the gastrointestinal tract in an intimate unit between the host and the microbes, called the holobiont [1,2]. With over 1000 bacterial species encoding around 100-fold more genes than the human genome, it has remarkable metabolic and functional capacity [3,4]. Among the prevalent bacterial phyla, Bacillota and Bacteroidota [5,6] comprise more than 90% of the intestinal microbial community combined [7], and their ratio is a key marker for intestinal homeostasis [1]. The microbiota performs indispensable structural, protective, neurological, and metabolic roles that together form the basis of host health [1,3]. Its composition is actively influenced by dietary habits, age, antibiotic use, host genetics, geographic location, and environmental stress [1,8]. There is strong evidence of a correlation linking gut dysbiosis with reduced microbial diversity and a disruption in the balance of microbial communities, and inflammatory and metabolic disorders, including obesity and type 2 diabetes mellitus (T2DM) [1,9,10]. Beyond metabolic disease, dysbiosis plays a part in colorectal carcinogenesis, as well as in neuropsychiatric diseases such as Alzheimer’s disease and major depression, via the impairment of homeostasis of the gut–brain axis [1,9,11,12]. Pathological states, in turn, perpetuate dysbiosis, developing a self-amplifying cycle of deteriorating disease outcomes and diminished therapeutic responses [1,10,13]. These two-way relationships have established the GM at the forefront of the modern research into chronic disease prevention and management [9,14]. Among all the dietary matrices, dairy products have a privileged position as precision modulators of GM, as they have a uniquely complex bioactive architecture, which is the conceptual foundation for this review.

### 1.2. Dairy Products as Functional Modulators

Milk and its products are an exceptionally rich matrix of bioactive compounds, and they include WP (LF, α-LA, LZ, and lactoperoxidase), caseins (CN), and their BP derivatives, GMP, and MOs [1,15]. These molecules exhibit collective antimicrobial, immunomodulatory, anti-inflammatory, and antitumorigenic characteristics, making dairy products functional foods with wide therapeutic potential [1,16,17]. The prebiotic action of MOs and WP selectively stimulates the growth of the beneficial genera, especially *Bifidobacterium* and *Lactobacillus*, with the aim of restoration of health-promoting GM in both infants and adults [1,18]. Fermented dairy products, including but not limited to yogurt, kefir, and other cultured milk beverages [19], further provide live probiotic strains together with fermentation-derived postbiotics (compounds that stimulate the growth of other types of butyrate- and propionate-producing flora) [20], including short-chain fatty acids (SCFAs), BPs, exopolysaccharides (EPSs), and bacteriocins (Bac) [20,21]. SCFAs interact with G-protein-coupled receptors (GPCRs), increase expression of tight junction proteins (ZO-1, occludin), and activate the immune system via TLR2/4 signaling pathways [9,22,23], promoting the integrity of the epithelial barrier and reducing intestinal inflammation [20,23,24]. At the molecular level, milk-derived β-CN peptides fine-tune the activity of macrophages through crosstalk between TLR, NF-kB, and MAPK signaling pathways that provide a mechanistic basis of dairy-mediated immunomodulation [25,26,27]. Food-derived BPs have the benefits of low toxicity, high biocompatibility, and various physiological activities when compared to synthetic therapeutic agents [27,28,29,30]. Meta-analyses of clinical interventions corroborate the results of regular yogurt or kefir intake, showing significant decreases in fasting glucose, low-density lipoprotein (LDL) cholesterol, and systemic C-reactive protein (CRP) in dysbiosis-associated conditions, with effect sizes dependent on strain composition, dose, and host genotype [31,32,33,34].

### 1.3. Precision Nutrition Framework and Objectives

**Figure 1:**
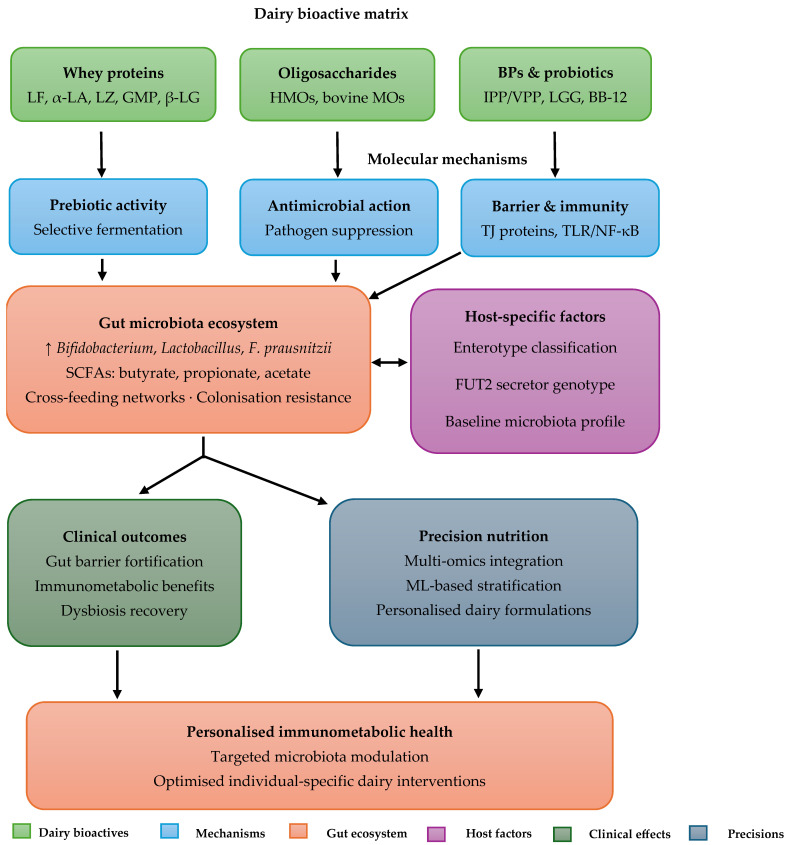
Conceptual framework illustrating dairy bioactive compounds as precision modulators of gut microbiota for personalized immunometabolic health. Dashed bidirectional arrow (⟷) between the gut ecosystem hub and host-specific factors reflects the reciprocal interaction between microbiota state and host determinants. LF, lactoferrin; α-LA, α-lactalbumin; LZ, lysozyme; GMP, glycomacropeptide; β-LG, β-lactoglobulin; HMOs, human milk oligosaccharides; BPs, bioactive peptides; LGG, *Lactobacillus rhamnosus* GG; BB-12, *Bifidobacterium animalis* subsp. *lactis* BB-12; IPP/VPP, Ile-Pro-Pro/Val-Pro-Pro; TJ, tight junction; TLR, toll-like receptor; NF-κB, nuclear factor kappa B; SCFAs, short-chain fatty acids; FUT2, fucosyltransferase 2; ML, machine learning.

Despite the compelling evidence for the DB components as GM modulators, three critical knowledge gaps remain, including dose–response characterization, long-term clinical efficacy, and elucidation of the mechanism’s pathway [9,35,36]. Most clinical trials are still of short duration, of four to twelve weeks, limiting understanding of the strength of the microbial changes and consequent immunometabolic effects [9]. Furthermore, host-specific determinants, such as baseline enterotype classification, FUT2 secretor genotype, and individual metabolic phenotype [9,37], present significant interindividual variability that cannot be adequately covered by recommendations at the population level [9]. The precision nutrition paradigm directly tackles this challenge by linking multi-omics data, i.e., metagenomics, metabolomics, and transcriptomics, with host genetic profiling to tailor dietary interventions to the individual microbiome ecosystems [35,38,39]. This framework allows predicting microbiota biomarkers to be identified, intervention responders from non-responders to be separated, and specific dairy-based formulations to be rationally designed for specific immunometabolic outcomes [9,35]. This review addresses critical knowledge gaps in dose–response relationships, long-term efficacy, and mechanistic pathways and suggests future directions for precision nutrition. By modifying molecular mechanisms in clinical applications, we have identified DB compounds as promising candidates for targeted modulation of the microbiota to optimize health and disease management. These concepts underpin a precision nutrition approach where DBs shape the GM via prebiotic, antimicrobial, barrier-enhancing, and immunometabolic mechanisms, and have varying effects depending on individual host characteristics, including enterotype, FUT2 genotype, baseline microbiota composition, gastrointestinal transit, and luminal pH (Figure 1).

## 2. Dairy Bioactive Compounds Classification and Sources

### 2.1. Whey Protein Fractions

Milk contains two main classes of proteins, which are CNs (around 80%) and WPs (around 20%), the latter of which is released in the soluble fraction after the coagulation of CNs [1,21]. Human WPs are composed of α-LA, LF, LZ, lactoperoxidase, GMP, immunoglobulins, and serum albumin (SA), while the bovine whey contains β-lactoglobulin and lower levels of α-LA [1,40]. These proteins collectively have antioxidant, anticancer, antimicrobial, anti-inflammatory, and immunomodulatory activities, with increasing evidence implicating these proteins in the modulation of the GM in both infants and adults [1,17,41]. LF, an 80 kDa iron-binding glycoprotein belonging to the superfamily of transferrins, is especially specific to colostrum and has dual direct and indirect prebiotic effects, i.e., it delivers β-N-glycans and iron for the growth of *Bifidobacterium* [42,43]. Meanwhile, iron sequestration function inhibits the growth of selectively (iron-independent probiotics, such as *Lactobacillus* and *Bifidobacterium*) [44,45,46]. α-LA is a 14 kDa globular protein that constitutes around 35% of human and 17% of bovine WPs [1,47,48]. LZ is a 14.4 kDa antibacterial enzyme that is particularly concentrated in human colostrum (0.2–0.9 g/L) [49,50]. These proteins selectively inhibit enteric pathogens and selectively spare probiotic strains of bifidobacteria by non-enzymatic mechanisms [1,15]. GMP, a 64-amino acid-long glycosylated peptide obtained by the cheese-making process from k-CN, contains sialic acid-rich oligosaccharide chains [51] that are responsible for high bifidogenic prebiotic properties, promoting the growth of *Bifidobacterium infantis*, *B. breve*, and *B. bifidum* [52,53].

### 2.2. Milk Oligosaccharides

MOs are a structurally diverse family of non-digestible carbohydrates assembled from five basic sugar residues of glucose, galactose, N-acetylglucosamine, fucose, and sialic acid, with various degrees of polymerization [54,55]. They are grouped in three structural classes: fucosylated (Fuc), non-Fuc neutral, and sialylated oligosaccharides [56]. Human milk (HM) has about 20-fold higher concentrations of oligosaccharides than bovine milk (BM), with Fuc and neutral oligosaccharides dominating in HM [57,58], whereas they are dominated by sialylated oligosaccharides in BM [57,59]. More than 200 different human MO structures have been identified, making them the third most abundant solid component of HM [60,61]. MOs have a prebiotic activity through two complementary and mutually reinforcing mechanisms. First of all, they are selectively fermented by *Bifidobacterium* strains, with examples including *B. infantis*, *B. breve*, and *B. bifidum* [62], and some strains of *Lactobacillus*, promoting bifidogenic changes in the microbiota composition [63,64]. Second, Fuc MO classes function as free receptor analogs that competitively block epithelial attachment by enteric pathogens [65] such as *Vibrio cholerae*, *Salmonella fyris*, *Campylobacter jejuni*, *Clostridioides difficile*, as well as *Escherichia coli* strains [66,67]. Through Bifidobacterium-mediated fermentation, MOs promote the stimulation of SCFA, especially butyrate production [1], as well as the functioning of the mucosal barrier of the gut, as well as the production of neuroactive metabolites, like for example the production of indole-3-lactic acid [68,69] or the neurotransmitter gamma-aminobutyric acid (GABA), linking the intake of oligosaccharides to the homeostasis of the gut–brain axis [1,70].

### 2.3. Bioactive Peptides and Probiotics

Dairy BPs are encrypted in the sequences of the milk proteins (mainly CNs and WPs) and released through the processes of enzymatic hydrolysis, gastrointestinal digestion, or microbial fermentation [21,71]. Food-derived BPs have a special therapeutic appeal because of their low toxicity, high biocompatibility, and a wide variety of physiological activities compared to synthetic drugs [28,72,73]. Among the most studied are IPP (Ile-Pro-Pro) in addition to VPP (Val-Pro-Pro) tripeptides released from β-CN and κ-CN by lactic acid bacteria (LAB) that inhibit angiotensin-converting enzyme (ACE) potently at millimolar concentrations, and result in clinically significant reductions in blood pressure [74,75,76]. The bovine β-CN-derived peptide YPFPGPIH is an emerging candidate with multifaceted immunomodulation of macrophages via crosstalk between TLR2/4, NF-kB, and MAPK signaling pathways at concentrations as low as 25–100 µM [27]. Probiotics in fermented dairy matrices or delivery forms, examples including *Lacticaseibacillus rhamnosus* GG (LGG), *Bifidobacterium animalis* subsp. *lactis* BB-12, as well as *Streptococcus thermophilus*, are another major bioactive type [3,25,77]. Fermented dairy matrices are protective vehicles buffering gastric acid, a source of prebiotic substrate that improves the engraftment of probiotics, and a source of CNs and WPs, as well as calcium and phospholipids, that allow maintenance of viability and mucosal colonization of probiotics [3,9]. Probiotic strains can be in the gut for days to weeks at a time, producing the postbiotic metabolites SCFAs, EPSs, and bacteriocins, all of which increase the expression of tight junction proteins (ZO-1, occludin) and modulate TLR2/4-mediated immune signaling [78,79,80]. The synergistic effect of peptides, oligosaccharides, and live probiotics through the dairy matrix forms a multi-target bioactive system, which is uniquely suited for precision modulation of the GM [1,9,81]. A structured summary table of the principal DB compounds, structural features, and major GM modulation mechanisms is shown in Table 1.

## 3. Molecular Mechanisms of Microbiota Modulation

### 3.1. Prebiotic Effects and Selective Fermentation

DB compounds affect GM by two mechanistically distinct prebiotic pathways: direct selective provision of substrates and indirect reshaping of the environment [1,9]. MOs are paradigmatic direct prebiotics; they make it intact into the colon and are selectively fermented by specific strains of Bifidobacterium bacteria, mainly *B. infantis*, *B. breve*, and *B. bifidum* [93], due to their unique repertoire of glycoside hydrolases and carbohydrate-binding molecules [94,95]. Structural variation among the MO classes determines their substrate specificity: Fuc oligosaccharides (e.g., 2’-O-fucosyllactose) are preferentially consumed by *B. infantis* [96] and sialylated species by different subpopulations of bifidobacteria, thus determining community composition with molecular precision [1,91,97]. WPs support in a parallel way: the LF is responsible for the supply of β-N-glycans that stimulate the growth of bifidobacteria and, separately, the iron to support the proliferation of *B. breve* by its holo form [1,44,98,99]. LF’s prebiotic activity goes as far as the transcriptional level; LGG treatment with bovine LF (1 mg/mL) was found to increase expression of genes involved with ABC transporter permeases, amino acid synthesis, DNA replication, and peptidoglycan biosynthesis [1,100]. While it was also found to downregulate the expression of genes involved with purine and pyrimidine catabolism pathways, revealing a genome-wide metabolic reprogramming of probiotic activity [1,100]. GMP has prebiotic effects by means of its sialic acid-rich oligosaccharide chains that selectively stimulate *B. infantis*, *B. breve*, and *B. bifidum* [101]. Moreover, another recent in vivo study in mice showed simultaneous increases in *Lactobacillus* and *Bifidobacterium* and significant decreases in fecal *Enterobacterales* and *coliforms* [1,87]. α-LA hydrolysates enhance SCFA-producing bacterial taxa, reducing pathobiont-associated genera, as shown in hyperuricemic mice [83], linking the prebiotics, or health effects, of WPs to downstream metabolite effects [1,102]. The fermentation activity of dairy-associated strains, including *Lactobacillus*, *Bifidobacterium*, and *Streptococcus*, was found to produce millimolar concentrations of acetate, propionate, and butyrate [9,103,104]. SCFAs activate G-protein-coupled receptors FFAR2 and FFAR3, inducing upregulation of TJ proteins (ZO-1, occludin), mucus secretion, and TLR2/4 immune signaling—all contributing to tight mucosal epithelial barriers [9,105,106]. Propionate, through the FFAR3 receptor on immune cells, specifically inhibits IL-6 and TNF-α secretion, providing targeted anti-inflammatory protection [107,108,109]. There is clinical support for these mechanisms in 28 patients with IBS who were administered *Bifidobacterium*-containing fermented dairy products daily; the fecal SCFA production and butyrate increased significantly after 11 days in conjunction with a decrease in a specific type of pathobiont called *Bilophila wadsworthia* and improvement in IBS symptoms [110]. These prebiotic and fermentative mechanisms are not independent but interact with one another in such a way that enables the enrichment of beneficial taxa [111]. Moreover, their SCFA products amplify concurrently the antimicrobial effects and interactions (cross-feeding) described in the subsequent sections, indicating a modulatory network rather than a collection of independent effects [112].

### 3.2. Antimicrobial Activities and Pathogens Suppression

DB compounds inhibit pathogens in the gut by overlapping direct and indirect molecular mechanisms that re-establish colonization resistance [9,113]. LF deploys two complementary antimicrobial pathways: iron sequestration that starves iron-dependent pathogens such as *E. coli* and *Staphylococcus aureus* [114]. Furthermore, it shows bactericidal activity from binding and neutralization of anionic bacterial surface constituents, e.g., lipopolysaccharide (LPS), through its very positively charged N-terminal domain [82,115]. This neutralization of LPS suppresses the TLR4 signaling pathway and the downstream NF-kB/MAPK/Nrf2 activation pathway to restrict the systemic endotoxemia and intestinal inflammatory pathways [116,117]. Its derivative Lactoferricin further interferes with the cell membrane integrity of pathogens by pore formation, expanding the antimicrobial spectrum [118,119]. LZ has a bacteriolytic action due to a breakage of the β-1,4-glycosidic bond between N-acetylmuramic acid and N-acetylglucosamine in peptidoglycan [84,120]. This is leading to rapid lysis of Gram-positive pathogens [85], whereas its non-enzymatic membrane-disrupting activity gives this molecule a partial activity against Gram-negative organisms [85,120]. Crucially, probiotic strains of the genus *Bifidobacterium* have evolved mechanisms to resist killing by LZs (mainly surface layer modifications) [86], so that the beneficial microbiota are spared, but the pathogens are suppressed [121,122]. LAB, which resides in the fermented dairy matrices, and *Lactobacillus*, *Bifidobacterium*, and *Streptococcus* species add another layer by producing narrow- to broad-spectrum bacteriocins [91,92]. Nisin of *Lactococcus lactis* provides lethal membrane pores and 8 log units reduction in the counts of E. coli O157:H7 in ripened cheddar in 60 days [123,124,125]. *Limosilactobacillus reuteri* produced reuterin, which interferes with quorum-sensing-dependent biofilm formation of *Salmonella* spp. [126], whereas glycoproteins and peptides from *Lacticaseibacillus casei* Shirota interfere with *C. difficile* biofilms by more than 60% in colonic mucus models [127]. At the ecological level, the probiotic strains (e.g., *Lactobacillus* and *Bifidobacterium*) outcompete *E. coli* and *Salmonella* for host-derived glycans and dietary fibers and occupy mucin receptors (MUC2) and epithelial niches, creating colonization resistance through niche pre-emption [127]. Collectively, these mechanisms are synergistic: in murine models of colitis, administration of *Li. reuteri* KUB-AC5, combined with the effects of ecological exclusion, resulted in significant attenuation of Salmonella-induced inflammation and pathogen burden [128]. In another clinical study, the daily consumption of kefir of 300 mL/day resulted in 40% decreased levels of fecal *E. coli* and serum levels of the protein Zonulin (by 18%) in IBS patients, quantitatively linking antimicrobial mechanisms to quantifiable improvements in gut barrier integrity [129]. These antimicrobial properties are in addition to the prebiotic effects of probiotics on beneficial colonizers (Section 3.1), as the ecological dominance of the probiotics also limits the ability of pathogens to find niches for nutrition and adhesion, and the cross-feeding substrates of acetate and lactate provide metabolic resilience at the community level (Section 3.3).

### 3.3. Microbial Cross-Feeding and Community Dynamics

Cross-feeding networks are at the heart of the community-level impact of DB compounds and translate temporary microbial stimulation into long-lasting ecosystem restructuring [3,130]. The main cross-feeding axis triggered by dairy prebiotics includes the bifidogenic fermentation of MOs and glycans of WPs producing acetate and lactate [131]. These are, in turn, consumed by specialized anaerobes of the colon, i.e., *Anaerostipes* spp., *Eubacterium hallii*, and *Coprococcus catus*, which improve intermediates to butyrate via butyryl-CoA: acetate CoA-transferase pathways [9,132]. In these lactate-utilizing bacteria, lactic acid acts as an electron sink, facilitating NAD+ regeneration to maintain the thermodynamics of fermentation and resulting in the metabolic exchange network [133]. Remaining lactose residues from dairy products fermentation reaching the colon contribute extra substrate to fuel this cross-feeding cascade to boost total SCFA output beyond what would be produced by direct fermentation alone [9,134]. The ecological consequences are quantifiable. In professional female soccer players, a 28-day course of kefir (200 mL/day) increased both Shannon and Chao1 diversity indices along with increases in the abundance of *Akkermansia muciniphila* and *Faecalibacterium prausnitzii*, keystone butyrate producers and mucosal barrier reinforcers [135]. This demonstrates that dairy intake restructures community membership in functionally meaningful ways [136]. Similarly, the consumption of kefir of 180 mL/day for twelve weeks by adults with metabolic syndrome increased the relative abundance of *Actinomycetota*, suggesting phylum-level changes that were consistent with re-established eubiotic balance [137]. Mutualistic inter-strain interactions within the dairy matrix itself further enrich the community dynamics [138]. The well-characterized *S. thermophilus* and *Lactobacillus delbrueckii* subsp. *bulgaricus* mutualism is a good example of this type of interaction: *L. delbrueckii* subsp. *bulgaricus* is responsible for the hydrolysis of CNs, which are digested to yield peptides and amino acids that feed *S. thermophilus* [139]. *S. thermophilus*, in return, supplies the host with formate and folate to stimulate the growth of its partner and improve metabolite biosynthesis [140,141]. These inter-species metabolic dependencies formed with engineering expand the spectrum of SCFA and strengthen community resilience [142]. Supplementation of the microbiota of Crohn’s disease patients with butyrate-producing bacteria, including *F. prausnitzii* and six butyrate producers, was found to increase the production of butyrate and restore epithelial barrier integrity in vitro [23,143]. The results of this study offer direct causal evidence that butyrate production by cross-feeding supports the intestinal barrier and inhibits pro-inflammatory NF-kB signaling [144]. Collectively, the prebiotic enrichment of keystone taxa (Section 3.1), the antimicrobial repression of competing pathogens (Section 3.2), and the cross-feeding metabolic networks described here constitute a triad of mechanisms that form an integrated architecture by which DB compounds enable lasting community-wide restructuring of the GM across ecological, metabolic, and structural lines [1,3,9].

## 4. Intestinal Barrier and Immunometabolic Regulation

### 4.1. Barrier Fortification: Tight Junctions and Mucus Layer

The intestinal epithelial barrier (IEB) is a single-cell layer defense system that physically separates luminal microbiota and antigens from the mucosal immune compartment [145,146]. Its integrity relies on the coordinated expression of tight junction (TJ) proteins—principally ZO-1, occludin, and claudins, which seal the paracellular space and regulate the transepithelial permeability [147,148]. DB compounds strengthen the integrity of the barriers by direct protein-mediated and metabolite-mediated mechanisms [149,150]. LGG-derived soluble proteins p40 and p75 maintain the integrity of Caco-2 TJs under stress by cytokines [9,90,151]. This is through the activation of the epidermal growth factor receptor (EGFR)/Akt signaling pathway via the ERK1/2 and PKCb1 pathways, i.e., direct prevention of the increase in paracellular permeability [152,153]. The LGG-secreted protein HM0539 inhibits the TLR4/MyD88/NF-kB pathway in colonic tissues, which normally has a pro-inflammatory mediator production that interferes with TJ assembly [88,89]. EPS of LAB, fermented whey, and dairy-derived lactic acid also lead to enhanced expression of ZO-1, occludin, and claudin-1 in colonic epithelial models, which can also restore the barrier function during inflammatory challenge [9,154]. Kefir enriches *Akkermansia muciniphila*, a mucin-degrader commensal whose colonization paradoxically thickens the mucus layer and improves mucosal barrier function and reduces the permeability of the intestinal barrier linking the community shifts mediated by dairy to structural reinforcement of the barrier [129,135,155]. Fatty acids for fermented milk (using *S. thermophilus*, *L. delbrueckii* subsp. *bulgaricus*, as well as *Lactiplantibacillus plantarum* A3) ameliorated DSS-induced colitis in mice through affecting the abundance of *Akkermansia* and *Lactobacillus*, decreasing the expression of IL-6, TNF-α, and inhibiting the phosphorylation of JNK in the mitogen-activated protein kinase (MAPK) pathway [156]. Clinically, the improvements in the barrier function seen with SCFA production are correlated with the 20–30% reduction in LPS levels in the circulation in cohorts with metabolic dysfunction, which provides a quantitative measurement of the barrier restoration [157,158].

### 4.2. Immune Cell Manipulation and Cytokine Networks

DB compounds have multiple interactions with the mucosal and systemic immune systems by multi-target modulation of innate and adaptive immune cell populations [159]. The major sentinels of innate immunity, macrophages, are specifically reprogrammed by milk-derived peptides [160]. The bovine β-CN peptide YPFPGPIH increased macrophage proliferation and phagocytosis in a dose-dependent manner (25–100 uM) and chemotactic migration by upregulation of monocyte chemoattractant proteins MCP-1 and MCP-3 [27]. Under LPS-stimulated inflammatory conditions, YPFPGPIH decreased IL-1β and TNF-α production, and at the same time enhanced IL-10 production, thus restoring the homeostasis of the cytokines [27,161]. Mechanistically, it suppressed NF-kB nuclear translocation (reduced p65) and reduced phosphorylation of ERK, JNK, and p38, effectively suppressing iNOS expression and nitric oxide overproduction [162,163]. This TLR2/4-mediated multi-pathway suppression is evidence that milk peptides can fine-tune the polarization of macrophages without inducing immunosuppression [27,164]. k-CN fragments produced by *S. thermophilus* promote the secretion of IL-10 in dendritic cells, promoting regulatory T-cell differentiation and reducing mucosal inflammatory responses [9]. The β-CN-derived peptide BCCY-1 activates TLR4/NF-kB in order to promote IL-10, forming an autocrine anti-inflammatory feedback loop [165]. At the adaptive immune level, Bifidobacterium-mediated immune imprinting mediated by MO-induced bifidogenic shifts helps to reduce allergy risk, asthma susceptibility, and systemic inflammatory conditions in infants based on the modulation of the regulatory T-cell/effector T-cell balance [166]. A meta-analysis of twelve RCTs (*n* = 684) found supplementation of T2DM patients with probiotic dairy resulted in a decrease in CRP of 1.34 mg/L (1.76 to 0.92), proving the systemic anti-inflammatory effects [167].

### 4.3. Short-Chain Fatty Acid-Mediated Immunometabolic Signaling

SCFAs, including acetate, propionate, and butyrate, produced during dairy fermentation, are the main metabolic currency linking microbiota activity and systemic immunometabolism regulation (IM) [23,168]. Butyrate serves as a ligand for GPR41, while propionate serves for both GPR41 and GPR43 on epithelial and immune cells [112]. These stimulate intracellular cascade signaling, which inhibits histone deacetylases (HDACs), resulting in increased histone acetylation [169,170] and transcriptional activation of TJ-encoding genes (ZO-1, occludin) [171]. Propionate acting through FFAR3 on circulating immune cells has the specific effect of inhibiting the secretion of IL-6 and TNF-α, and this provides targeted anti-inflammatory action at the periphery [22,23]. Butyrate-enriched fermented milk also has additional anti-inflammatory properties that target colonic NF-kB activation beyond single pathway activation [9,172]. On the metabolic side, the enrichment of yogurt and kefir with conjugated linoleic acid was found to inhibit HMG-CoA reductase and upregulate hepatic LDL receptors, reducing the circulating LDL cholesterol [173,174]. Kefir administration in mice fed with a high-fat diet decreased their body weight and hepatic lesion scores and enhanced the fatty acid oxidation gene expression (PPARa) in liver and adipose tissue [175]. In addition to decreasing the circulating IL-6 concentration, links are made between dairy-derived SCFA signals and hepatic lipid homeostasis [176,177]. In controlled clinical trials, administration of 300 g/day probiotic yogurt (*L. acidophilus* La5 and *B. animalis* subsp. *lactis* Bb12) for 6 weeks reduced the LDL cholesterol level by 7.45% and total cholesterol levels by 4.54%; these are dose-dependent glycemic and lipid benefits [178]. Kefir consumption has a significant glycemic-lowering effect, such as 95 mg/dL to 83 mg/dL in the fasting blood glucose levels in T2DM patients [179], and for its mechanism, the engagement of SCFA on GLP-1 secretion pathways is proposed [23,180,181]. These SCFA-mediated IM effects are dose- and strain-dependent, which not only highlights the precision nutrition imperative of tailoring dairy formulations to individual metabolic phenotypes [23].

### 4.4. Multi-Axis Communications (Gut–Brain, Gut–Liver)

DB compounds exert their IM effects in the extra-gut via bi-directional multi-organ communication axes [182,183,184,185]. Along the gut–brain axis, the supplementation of kefir increases the growth of fecal butyrate-producing taxa such as *Lachnospiraceae* and *Lachnoclostridium* [186]. Moreover, it modulates the production of SCFA, in particular butyrate and propionate, both in colon and brain tissue, leading to better outcomes in brain health [186]. In a mouse model of autism, reshaping the microbiota improved immune function and diminished repetitive stereotyped behaviors, proving that there is a causal link between the microbiota, immune system, and brain [187]. GABA-enriched fermented milks increase circulating as well as brain concentrations of GABA, leading to improved mood and cognition in aging rodents, through modulating PI3K/AKT/mTOR and GABAB-cAMP-PKA-CREB signaling [9,188,189,190]. In another human study, the administration of yogurt and cheese is associated with significantly reduced anxiety scores on the Spielberger anxiety State-Trait Anxiety Inventory in university students [191]. This suggests translational evidence for the effects of dairy-derived bacterial activity in the production of the neurotransmitter GABA [9,192]. Bifidobacterium-induced synthesis of indole-3-lactic acid was found to involve the activation of aryl hydrocarbon receptor (AhR) [193], which is the regulator of gut–brain axis homeostasis, intestinal immune response, and epithelial integrity, and therefore connects MO fermentation to neuroimmune signaling [68,194]. Along the gut–liver axis, the dairy-derived SCFAs are delivered to the portal circulation by the gut–portal route [195]. This is where they are used by the liver as a gluconeogenic substrate (propionate) and as an activated receptor (PPARa) substrate to regulate hepatic lipid oxidation [196,197], which leads to the reduction in ectopic fat accumulation [198]. Fermented milk peptides orally administered by aged mice, in particular, WY dipeptide (10 mg/kg, 14 days), inhibit LPS-induced activation of hippocampal microglia and enhance long-term potentiation (LTP), showing the cross-barrier effect of DB components against neuroinflammation [199]. DB compounds, besides being primarily considered as gut effectors, are thus positioned as systemic IM modulators that make them especially attractive candidates for precision interventions against neuro-metabolic comorbidities [182,200].

## 5. Precision Nutrition: Host-Specific Response

### 5.1. Enterotype and Genetic Determinants

Host enterotype and genetic background are shaped by various individuals’ responsiveness to the DB interventions, so population-level recommendations are inadequate [159,201]. Enterotypes, defined as different community-state types dominated by *Bacteroides*, *Prevotella*, or *Ruminococcus*, impose tremendously different metabolic capacities on the gut ecosystem [202]. This subsequently modulates the magnitude of dairy-induced microbiota shifts [201,202]. Bacteroides-dominant enterotypes, for example, have been associated with increased production of SCFA after consumption of kefir, reflecting the role of community architecture in determining metabolic outcomes following identical dietary inputs [203,204]. The FUT2 “secretor” genotype has been the most characteristic and clinically described genetic determinant of response to probiotics [205]. Secretor status is determined by one of its enzymes, functional α 1,2-fucosyltransferase 2 (FUT2), which controls the expression of Fuc mucosal glycans, which act as substrates and adhesion receptors for commensals [205]. FUT2 secretors have a significantly higher baseline Bifidobacterium diversity and abundance, which results in a higher enrichment by fermented dairy intake [205]. On the other hand, FUT2 non-secretors (homozygous for inactivating alleles such as rs601338 G428A) have much lower concentrations of Bifidobacterium spp., which results in a compromised ability to ferment prebiotic oligosaccharides in dairy matrices and lower SCFA production and support for the intestinal barrier [205,206]. Beyond FUT2, more general host genetic variation contributes to the composition of the host microbiota across taxa and associated phyla [207], as shown by genome-wide association studies that relate host single-nucleotide polymorphisms to certain microbial phenotypes [208]. Gastrointestinal physiological features, transit time, luminal pH, and the mucosal glycosylation patterns contribute to interindividual variability that interacts with the genetic background to determine the probiotic colonization rate as well as metabolite patterns [9,209]. These findings together define that profiling of the genetic and enterotype before intervention is a prerequisite and not an optional refinement to design-efficacy dairy-based precision nutrition strategies.

### 5.2. Baseline Microbiota as Response Predictor

Baseline GM composition has become the single most predictive factor in individual responsiveness to DB interventions and is more predictive than static genetic markers [9,210,211]. This is because the resident microbiota influences which substrates may be fermented, which metabolic pathways are functionally active [212], and how much ecological space is available for exogenous probiotic strains to occupy [213,214]. Probiotic strains are individual-, site-, and strain-dependent in their colonization of the gut mucosa in a highly personalized way, and are influenced by each individual’s pre-existing microbial profile, not by fecal detection of ingested strains [211,215]. Post-antibiotic probiotic supplementation failed to restore the microbiome in a global manner [216] but showed significant host dependence, which directly showed the role of the baseline community state in governing the success of probiotic intervention [217,218]. Baseline microbiota profiling using α-diversity measures and enterotype classification can be used to stratify responders and non-responders to the intervention prior to the start of the intervention [210,215,219,220]. Recent trials have taken advantage of baseline functional gene clusters, specifically bile salt hydrolase (BSH) loci, to predict the metabolic improvements from fermented milk, linking pre-intervention microbiome function to clinical outcomes [9,210]. Similarly, baseline gut microbiome composition has been validated as a predictive biomarker for response to probiotic adjuvant treatment for the management of gout, establishing the concept of microbiome-stratified clinical trial design [211]. Intestinal physiological parameters, especially gastrointestinal transit time and colonic pH, have a major impact on the composition and metabolite profiles of the microbiota between individuals [221]. Hence, add a dynamic dimension of host-specific variation in addition to static baseline evaluations [221]. Of particular note, there are studies analyzing yogurt consumption by 16S rRNA in addition to the whole genome metagenomic data from the Twins UK cohort (*n* = 1004), which have revealed that host and environmental factors, rather than the dairy intervention itself, were the main drivers of variation in β-diversity [222]. This highlights the importance of taking pre-existing community structure into account when designing and interpreting dairy intervention studies [223,224]. The key host-specific determinants that shape individual responsiveness to DB interventions are summarized in Table 2.

### 5.3. Personalized Intervention Strategies

To translate the information on enterotypes and baseline microbiota into actionable personalized dairy interventions, the use of an integrated framework spanning omics profiling, technological innovation, and adaptive clinical design is needed [9,35,38]. Multi-omics integration of metagenomics, metabolomics, host genomics, and transcriptomics enables the identification of predictive microbiota biomarkers for stratifying responders and for the choices of the appropriate strains, as well as the dose and matrix composition for individual host–microbe ecosystems [182,225,226]. Metagenomics provides taxonomic as well as functional blueprints through the identification of species-specific gene clusters that encode the biosynthesis pathways of SCFAs [182], while metabolomics quantifies the SCFA, bile acid, and indole derivative outputs, which mediate cross-kingdom signaling [9,182]. Machine learning frameworks such as random forest and mediation analysis are increasingly applied to integrate these high-dimensional datasets, enabling prediction of personalized dairy responses from baseline multi-omics profiles [227,228]. On the technological front, portable nano-sequencers with nanopore cores and smartphone connectivity are now possible to perform weekly home-based profiling of the gut microbiome, to support real-time adaptive dietary changes [9]. Embedding specific probiotic strains into specific prebiotic matrices layer-specifically through utilizing 3D printing of functional foods can also further promote targeted colonization and strain-specific metabolite generation [229]. The pairing of transient starters (*S. thermophilus*) with persistent probiotics (LGG) in complex matrices, such as kefir product, provides both immediate metabolic stimulation in addition to enduring microbiota remodeling, and is a blueprint for temporally layered personalized formulations [9,230]. Fermentation parameters such as pH, temperature, substrate ratios, and processing conditions are modifiable levers that allow the optimization of peptide yields and SCFA composition according to individual metabolic phenotypes [9,229]. By combining the classification of the enterotype, the genotype, the baseline characterization of the microbiome, and the design of a formulation using machine learning algorithms, precision dairy nutrition can go a long way from population-wide recommendations to truly individual-specific recommendations [9]. This can provide optimized gut ecosystem modulation, improved therapeutic efficacy, and improved long-term immunometabolic health outcomes [9,35,38]. To implement enterotype classification, FUT2 genotyping, and baseline microbiome profiling in clinical practice, a stepwise, structured approach is needed. Some individuals should be screened for enterotype and α-diversity via shotgun metagenomics [201], then screened for secretor phenotype via FUT2 genotyping, and finally functional profiled for BSH gene cluster activity and SCFA biosynthesis capacity before intervention [205]. FUT2 secretors with Bacteroides-dominant enterotypes were predicted to be most responsive to oligosaccharide-enriched dairy matrices, while FUT2 non-secretors or those with less diverse microbial communities respond to lactoferrin given at high doses in synbiotic matrices [37,205,206]. People with post-antibiotic microbiome collapse may need a restoration phase before standard probiotic dairy supplementation to get to a point where there is enough mucosal engraftment [216,217]. These stratification variables then could be combined within machine learning models with metabolomics-based SCFA profiles to rank order candidate dairy formulations and predict individual response, making baseline biomarkers a dynamic tool to guide intervention, and bringing precision dairy nutrition from theory to practice [35,227,228].

## 6. Clinical Evidence and Therapeutic Applications

### 6.1. Infants’ Nutrition and Development

The period from birth to 2.5 years is the most important time frame for the development of GM, and the formation at this time has a lasting impact on immune programming and metabolic health [1,3,231]. Breast milk provides the main orchestrator of this developmental process, delivering a triad of bioactive modulators, HMOs, LF, and α-LA, which subsequently influence the development of the neonatal microbiome [232,233]. Metagenomic studies have shown that breast-fed infants have a less diverse but functionally better microbiota with health-promoting *Bifidobacterium*, *Lactobacillus*, and *Staphylococcus*, as the predominant species, than formula-fed [234,235]. HMOs selectively enhance the colonization of the *B. infantis*, *B. breve*, *Bifidobacterium longum*, and *B. bifidum* that create a so-called bifidogenic microbiota that is responsible for immune imprinting and protection against risks of allergy and inflammatory diseases [236,237]. Bifidobacterium-mediated fermentation of the HMOs also produces the neurotransmitters, GABA and indole-3-lactic acid, which activate the aryl hydrocarbon receptor, to regulate early gut–brain axis homeostasis [193,238,239]. In randomized controlled clinical trials in very low birth weight (VLBW) neonates, the supplementation of bovine LF (100 mg/day), alone or in combination with LGG (6 × 10^9^ CFU/day), significantly reduced the incidence of late-onset sepsis (a severe complication of prematurity) by reducing colonization by pathobionts [240,241]. Recombinant human LF (talactoferrin, TLf; 150 mg/kg/12 h for 28 days) in preterm infants reduced the incidence of urinary tract infection and produced positive changes in the fecal microbiome, such as decreases in the incidence of Gram-positive pathogenic bacteria [241,242]. The addition of oligofructose at a concentration of 3.0 g/L to a milk protein (α-LA-enriched infant formula) led to a significant increase in fecal bifidobacteria compared to control formula [243]. This is demonstrating synergistic prebiotic effects of combined WP and oligosaccharide supplementation [1]. These clinical findings collectively make DB compounds an evidence-based candidate for neonatal microbiota programming and infection prevention in early life.

### 6.2. Metabolic Disorders and Inflammation

Fermented dairy products have been shown to have strong clinical evidence for their role in the management of T2DM, the metabolic syndrome, obesity, and systemic inflammation [9,244]. A recent meta-analysis study of six RCTs (*n* = 323) showed a significant decrease in fasting blood glucose (WMD = −10.28 mg/dL; 95% CI: −16.53 to −4.02; *p* = 0.001) and reduction in serum insulin (WMD = −2.87; 95% CI: −3.96 to −1.78; *p* < 0.00001) following 4–12 weeks of kefir consumption in patients with T2DM [245]. In a controlled trial of newly diagnosed T2DM males, 100% of the patients in the kefir plus metformin group experienced a significant decrease in both fasting blood glucose and HbA1c over metformin alone (*p* < 0.05), indicating the microbiota-mediated enhancement of glycemic control through upregulated GLUT4 translocation [246]. The consumption of probiotic yogurt at a concentration of 300 g/day containing *L. acidophilus* La5 and *B. animalis* subsp. *lactis* Bb12, 6 weeks, was found to decrease LDL cholesterol by 7.45% and total cholesterol by 4.54% in patients with T2DM, together with a reduction in the ratio of LDL/HDL cholesterol from 3.13 + 1.00 to 2.07 [247]. In a later meta-analysis study of 12 RCTs (*n* = 684), it was found that daily supplementation of probiotics in dairy products reduced C-reactive protein by 1.34 mg/L (95% CI: −1.76 to −0.92) in T2DM patients (i.e., substantial attenuation of systemic inflammation) [167]. In cohorts of metabolic syndrome, the 12 weeks of kefir (180 mL/day) significantly lowered fasting blood glucose from 95 + 9 to 83 + 8 mg/dL (*p* < 0.05), fasting insulin, HOMA-IR, TNF α, and IFN-γ, and increased the abundance of beneficial Actinobacteria [179,248]. In one large cross-sectional study, the higher cheese consumption was associated with significantly decreased risk of obesity (adjusted OR = 0.70; 95% CI: 0.51–0.96; *p* < 0.05) [249]. Mechanistically, these benefits are mediated by CLA-driven inhibition of the HMG CoA reductase, SCFA-mediated suppression of NF-kB mediated by GPR43, and microbiota-driven secretion of GLP-1, integrating multiple molecular pathways towards metabolic disease management [9,167].

### 6.3. Dysbiosis Recovery and Aging

DB compounds have shown clinical therapeutic potential in reversing pathological dysbiosis in such diverse diseases as chemotherapy-induced microbiome disruption, HIV-associated dysbiosis, antibiotic-induced community collapse, and age-associated microbial decline [15,41,159,250]. In a double-blind, placebo-controlled clinical trial in pediatric oncological patients undergoing chemotherapy, oral bovine LF (200 mg/day for two months) was shown to facilitate the eubiosis of the gut microbiome by controlling the proliferation of pathobionts (in particular, Enterococcus) and maintaining the abundance of intestinal health-associated taxa (such as *Akkermansia*) [250]. This is ultimately enhancing the health outcomes and response to therapy [1,250]. In patients with HIV on antiretroviral therapy, recombinant human LF (1500 mg twice daily) has not significantly affected the composition of GM but provided microbial community stability through time [251]. This suggests a role of microbial community stabilization rather than reconstruction in this setting [251]. In a model of dysbiosis of the intestinal microbiome induced by antibiotic use, the anti-inflammatory bacteria, including *Bacteroidaceae*, *Prevotellaceae*, and *Rikenellaceae*, were restored to normal levels by both native and iron-saturated bovine LF, establishing preclinical proof of concept for the dysbiosis-reversing capacity of bovine LF [252]. Aging is linked to a progressive reduction in the abundance of colonic *Bifidobacteria* (90%), and this is correlated with an increase in GI disorders, systemic inflammation, and impaired immunity [253,254]. In a double-blind, placebo-controlled study of healthy elderly women, oral bovine LF (1 g/day) was shown to induce an increase in *Holdemanella* in fecal microbiota, and its combination with active galactooligosaccharides also increased Bifidobacterium abundance, showing additive bifidogenic effects for an elderly population [255]. An oral supplementation of HMOs (2’FL and LNnT) in healthy adults for two weeks had an effect to elevate the relative abundance of *Bifidobacterium* up to >25% in some individuals, with a concomitant decrease in *Bacillota* and *Pseudomonadota*, which showed that MO-based intervention could induce juvenile-like bifidogenic states in elderly adults [256]. These results collectively place DB compounds in the position of clinically proven therapeutic tools in the management of dysbiosis at all ages, from chemotherapy-induced disruption of microbial communities in children to the age-related microbial deterioration in elderly people [250,252,253].

## 7. Challenges and Future Perspectives

### 7.1. Bioavailability and Standardization Issues

One of the major barriers to the translation of DB research to clinical practice is the variability in the bioavailability of key compounds among individuals and types of products [257]. BPs, LF, and MOs undergo gastrointestinal proteolysis, pH-dependent degradation, and matrix interactions that greatly decrease their effective luminal concentrations [258,259]. Post-fermentation processing used in food, such as heat treatment, spray drying, and pasteurization, can also further degrade the viability of probiotics as well as the stability of peptides [260]. However, pasteurized kefir still maintains EPSs-inducing IL-10, implying that non-viable postbiotics still retain some function [104,261]. The direct prebiotic activity of LF is highly strain-specific and influenced by the iron-saturation level, temperature, and dosage factors not often controlled in a consistent way in different studies [262]. Standardization of dairy matrices for clinical application requires the determination of minimum effective doses, optimal fermentation parameters (pH, temperature, substrate ratios), and validated biomarkers of bioactive delivery to the colon [1,9]. Addressing these gaps is crucial if dairy bioactivities are to be developed as reproducible, evidence-based therapeutic products.

### 7.2. Research Gaps and Clinical Translation

The most important methodological gap in the field is the reliance on overwhelmingly short-term clinical designs [9,229]. Over 80% of the published RCTs of dairy matrices fortified with probiotics use intervention periods of only 8–12 weeks, and this leaves unresolved questions as to whether the restructuring of microbiota continues after discontinuation or if chronic conditions develop favorably after long-term exposure [9,33]. Extended intervention–withdrawal cycles, such as six-month dosing followed by three-month washout, are required to determine the resistance of the microbiota and host metabolic memory [263]. Germ-free mouse models are also of mechanistic value, but have underdeveloped immune systems and abnormal intestinal structure, so the models are less relevant to model human dairy interventions [264]. Causal mechanisms between certain postbiotic metabolites and clinical endpoints are yet unvalidated; engineered consortia in humanized models and well-controlled crossover trials with standardized dairy matrices have to be performed to prove causality [9,229]. Furthermore, dose–response relationships for individual compounds, mainly LF, GMP, and specific HMO structures, are incompletely characterized in human subjects in different disease states [229,265].

### 7.3. Regulatory Considerations

The regulatory status of DB compounds as functional foods, medical foods, or nutraceuticals differs considerably from one jurisdiction to another, thus creating barriers to market authorization and clinical use [266,267]. Most dairy bioactivities do not yet have the quality of clinical evidence needed to make health claims at the drug level, and regulatory agencies require reproducible compositional standards that fermented products with batch-to-batch variability have great difficulty achieving [268,269]. Robust frameworks for data ownership, consumer microbiome reporting, and consent for precision nutrition platforms are underdeveloped [210,211,229]. Future regulatory pathways will need to incorporate multi-omics data together with traditional RCT data, create standardized compositional specifications for bioactive dairy fractions, and build adaptive approval frameworks that take into account the variability of host-specific responses to allow dairy bioactivities to progress from being traditional food ingredients to being targeted therapeutic matrices [9,35].

## 8. Conclusions

This review shows that DB compounds that include WPs, MOs, BPs, and postbiotics that are fermentation-derived represent multi-target precision modulators of the GM through mechanistically distinct prebiotic, antimicrobial, and cross-feeding pathways. Their downstream effects go far beyond the gut lumen and include strengthening of the integrity of the epithelial barrier, reprogramming of innate and adaptive immune networks, and control of systemic IM homeostasis through the gut–brain and gut–liver axes. Clinical evidence confirms the dose-dependent benefits in a range of metabolism disorders, neonatal microbiome programming, and dysbiosis recovery, with effect magnitude modulated by enterotype, FUT2 genotype, and baseline microbiota composition. Integration of metagenomics, metabolomics, and machine learning can now make the switch from population-level dietary recommendations to individualized dairy formulations possible. Addressing critical gaps in dose–response characterization, long-term trial design, and regulatory standardization remains critical. Finally, DB compounds are evidence-based candidates for targeted microbiota modulation in personalized IM health management.

## Figures and Tables

**Table 1:** Classification, structural features, and gut microbiota modulation mechanisms of key dairy bioactive compounds.

Compound	Structural Features	Primary GM Modulation Mechanism	Key Microbial Taxa Affected	Selected References
Lactoferrin (LF)	80 kDa iron-binding glycoprotein; transferrin superfamily; enriched in colostrum.	Bifidogenic via β-N-glycan and iron delivery; iron sequestration to starve pathogens; LPS neutralization via positively charged N-terminal domain.	↑ *Bifidobacterium*, ↑ *Lactobacillus*; ↓ *E. coli*, ↓ *Staphylococcus aureus*	[42,43,44,82]
α-Lactalbumin (α-LA)	14 kDa globular protein; ~35% of human WP; ~17% of bovine WP.	Hydrolysates enhance SCFA-producing taxa; reduce pathobiont-associated genera; demonstrated in hyperuricaemic mouse models.	↑ SCFA-producing bacteria; ↓ pathobionts	[47,83]
Lysozyme (LZ)	14.4 kDa antibacterial enzyme; 0.2–0.9 g/L in human colostrum.	Bacteriolysis via β-1,4-glycosidic bond cleavage in peptidoglycan; non-enzymatic membrane disruption; *Bifidobacterium* spared via surface layer resistance mechanisms.	*Bifidobacterium* spared; ↓ Gram-positive pathogens	[49,84,85,86]
Glcomacropeptide (GMP)	64-amino acid glycosylated peptide from κ-CN; sialic acid-rich oligosaccharide chains.	Selective bifidogenic stimulation via sialic acid; reduces fecal *Enterobacterales* and *coliforms* in vivo.	↑ *B. infantis*, *B. breve*, *B. bifidum*; ↓ *Enterobacterales*, *coliforms*	[52,53,87]
Human milk oligosaccharides (HMOs)	>200 structurally distinct compounds; third most abundant solid in human milk; 3 classes: fucosylated, neutral, sialylated.	Selective fermentation by *Bifidobacterium* via unique glycoside hydrolase repertoire; anti-adhesion receptor decoys for enteric pathogens; SCFA production via cross-feeding.	↑ *B. infantis*, *B. breve*, *B. bifidum*, *Lactobacillus*; ↓ *V. cholerae*, *Salmonella*, *C. difficile*, *E. coli*	[60,62,65,66]
Bovine milk oligosaccharides (BMOs)	Dominated by sialylated oligosaccharides; 20-fold lower concentration than HMOs.	Prebiotic modulation distinct from HMOs; sialylated BMOs fermented by specific *Bifidobacterium* subpopulations.	↑ *Bifidobacterium* (sialylated-BMO-utilizing strains)	[57,59]
Bioactive peptides (BPs): IPP/VPP	Tripeptides (Ile-Pro-Pro, Val-Pro-Pro) from β-CN and κ-CN; released by LAB fermentation.	ACE inhibition at millimolar concentrations; blood pressure reduction.	Indirectly specified	[74,75]
β-CN peptide YPFPGPIH	Bovine β-CN derived; active at 25–100 μM.	Macrophage reprogramming via TLR2/4–NF-κB–MAPK crosstalk; ↑ IL-10, ↓ IL-1β, ↓ TNF-α; suppresses iNOS/NO overproduction.	Indirect: Macrophage polarization modulating mucosal immune environment	[27]
Probiotics: LGG, BB-12, *S. thermophilus*	Live microbial strains; fermented dairy matrix provides gastric acid buffering and prebiotic substrate.	Tight junction upregulation (ZO-1, occludin) via p40/p75/EGFR–Akt and HM0539/TLR4–MyD88–NF-κB pathways; Bac production; colonization resistance via niche pre-emption.	↑ Beneficial commensals; ↓ *E. coli*, *Salmonella*, *C. difficile*	[9,88,89,90,91,92]

WP, whey protein; CN, casein; GM, gut microbiota; iNOS, inducible nitric oxide synthase; LPS, lipopolysaccharide; SCFA, short-chain fatty acid; NF-κB, nuclear factor kappa B; ACE, angiotensin-converting enzyme; IL, interleukin; LAB, lactic acid bacteria; LGG, *Lactobacillus rhamnosus* GG; BB-12, *Bifidobacterium animalis* subsp. *lactis* BB-12; TLR, toll-like receptor; MAPK, mitogen-activated protein kinase; TNF-α, tumor necrosis factor-alpha; Bac, bacteriocin; ↑, increase; ↓, decrease.

**Table 2:** Host-specific determinants of individual response to dairy bioactive interventions: Implications for precision nutrition.

Determinant	Category	Mechanistic Basis	Consequence for Dairy Bioactive Response	Reference
Bacteroides-dominant enterotype	Microbial community architecture	Distinct carbohydrate-fermentation capacity and metabolic output compared to *Prevotella* or *Ruminococcus* types.	↑ SCFAs production following kefir consumption; community architecture determines metabolic magnitude of dairy-induced shifts.	[202,203]
Prevotella-dominant enterotype	Microbial community architecture	Different substrate utilization pathways and immune modulation capacity.	Divergent magnitude of dairy-induced microbiota shifts from Bacteroides-dominant individuals exposed to identical dietary inputs.	[201,202]
FUT2 secretor status (functional α1,2-fucosyltransferase)	Host genetics	Controls expression of fucosylated mucosal glycans that serve as colonization substrates and adhesion receptors for commensals.	Higher baseline *Bifidobacterium* diversity and abundance; greater microbial enrichment following fermented dairy or prebiotic oligosaccharide intake.	[37,205]
FUT2 non-secretor status (rs601338 G428A homozygous)	Host genetics	Inactivating alleles abolish mucosal fucosylation, removing commensal attachment substrates.	Significantly lower *Bifidobacterium* spp. abundance; compromised oligosaccharide fermentation; ↓ SCFAs production; impaired intestinal barrier support.	[205,206]
Baseline gut microbiota composition	Dynamic microbiome state	Determines ecological space available for probiotic engraftment; dictates which metabolic pathways are functionally active; governs community resilience to perturbation.	Single most predictive factor of individual responsiveness—more predictive than static genetic markers; probiotic colonization is highly personalized, site-dependent, and strain-dependent.	[210,211,215]
Post-antibiotic microbiome state	Baseline community disruption	Collapsed diversity and altered colonization resistance limit ecological scaffolding for probiotic engraftment; autologous FMT outperforms probiotic supplementation in restoration.	Post-antibiotic disruption reduces ecological scaffolding for probiotic engraftment and makes restoration highly host-dependent.	[216,217]
Bile salt hydrolase (BSH) gene cluster activity	Functional microbiome capacity	Encodes metabolic pathways predicting bile acid transformation and SCFAs biosynthesis capacity; functional blueprint beyond taxonomic profiling.	Baseline BSH locus activity predicts metabolic improvements from fermented milk consumption, linking pre-intervention microbiome function to clinical outcomes.	[210]
Host single nucleotide polymorphisms (SNPs, GWAS)	Host genomics	Genome-wide associations between host SNPs and specific microbial taxa and phenotypes.	Shapes community composition across taxa and associated phyla; genetic architecture of the host co-determines microbiome structure.	[207,208]
Gastrointestinal transit time	Physiological parameter	Modulates substrate availability and residence time for microbial fermentation; shapes microbial composition and metabolite profiles.	Adds a dynamic dimension to host-specific variation beyond static baseline or genetic evaluations; critical for interpreting intervention response variability.	[221]
Luminal colonic pH	Physiological parameter	Affects probiotic colonization rate, enzymatic fermentation activity, and the balance of acetogenic vs. butyrogenic pathways.	Contributes to interindividual variation in microbiota composition and metabolite profiles; interacts with genetic background to modulate probiotic colonization.	[209,221]
Mucosal glycosylation patterns	Physiological–genetic interface	Provides substrates and adhesion receptors for commensal bacteria; shaped by both FUT2 genotype and broader host genetics.	Determines ecological niches for commensals and probiotic strains, contributing to interindividual variation in colonization and metabolite patterns.	[205,209]
Pre-existing community diversity (α-diversity)	Microbiome ecological state	High-diversity communities provide enhanced colonization resistance; α-diversity at baseline determines ecological permissiveness for exogenous strains.	Baseline α-diversity and enterotype classification stratify responders from non-responders prior to intervention.	[215,219,220]

FUT2, fucosyltransferase 2; SNP, single nucleotide polymorphism; GWAS, genome-wide association study; BSH, bile salt hydrolase; SCFAs, short-chain fatty acids; FMT, fecal microbiota transplantation.

## Data Availability

No new data were created or analyzed in this study. Data sharing is not applicable to this article.

## Abbreviations

The following abbreviations are used in this manuscript:

BM	Bovine milk
IG	Immunoglobulin
LF	Lactoferrin
PGRP	Peptidoglycan recognition protein
LAB	Lactic acid bacteria
SA	Serum albumin
LA	Lactalbumin
CN	Casein
WP	Whey protein
LZ	Lysozyme
GABA	Gamma-aminobutyric acid
IM	Immunometabolism regulation
MOs	Milk oligosaccharides
BP/BPs	Bioactive peptide(s)
SCFAs	Short-chain fatty acids
T2DM	Type 2 diabetes mellitus
EPSs	Exopolysaccharides
Bac	Bacteriocin

## References

[1] Gallo V., Arienzo A., Tomassetti F., Antonini G. (2024). Milk bioactive compounds and gut microbiota modulation: The role of whey proteins and milk oligosaccharides. Foods.

[2] Thursby E., Juge N. (2017). Introduction to the human gut microbiota. Biochem. J..

[3] Abd El-Salam M.H., El-Shibiny S., Assem F.M., El-Sayyad G.S., Hasanien Y.A., Elfadil D., Soliman T.N. (2025). Impact of Fermented Milk on Gut Microbiota and Human Health: A Comprehensive Review: MHA El-Salam et al. Curr. Microbiol..

[4] Aggarwal N., Kitano S., Puah G.R.Y., Kittelmann S., Hwang I.Y., Chang M.W. (2022). Microbiome and human health: Current understanding, engineering, and enabling technologies. Chem. Rev..

[5] Oren A., Arahal D.R., Rosselló-Móra R., Sutcliffe I.C., Moore E.R. (2021). Emendation of Rules 5b, 8, 15 and 22 of the International Code of Nomenclature of Prokaryotes to Include the Rank of Phylum.

[6] Oren A. (2024). On validly published names, correct names, and changes in the nomenclature of phyla and genera of prokaryotes: A guide for the perplexed. NPJ Biofilms Microbiomes.

[7] Sarkar K., Sil P.C. (2021). Effect of diet, pharmaceuticals, and environmental toxicants on gut microbiota imbalance and increased intestinal membrane permeability. Toxicological Risk Assessment and Multi-System Health Impacts from Exposure.

[8] Dudek-Wicher R.K., Junka A., Bartoszewicz M. (2018). The influence of antibiotics and dietary components on gut microbiota. Prz. Gastroenterol..

[9] Gao Y., Liu Y., Ma T., Liang Q., Sun J., Wu X., Song Y., Nie H., Huang J., Mu G. (2025). Fermented dairy products as precision modulators of gut microbiota and host health: Mechanistic insights, clinical evidence, and future directions. Foods.

[10] Shen Y., Fan N., Ma S.X., Cheng X., Yang X., Wang G. (2025). Gut Microbiota Dysbiosis: Pathogenesis, Diseases, Prevention, and Therapy. MedComm.

[11] Lei W., Cheng Y., Liu X., Gao J., Zhu Z., Ding W., Xu X., Li Y., Ling Z., Jiang R. (2025). Gut microbiota-driven neuroinflammation in Alzheimer’s disease: From mechanisms to therapeutic opportunities. Front. Immunol..

[12] Sun X., Li Y., Li W., Koeban S., Zhang L., Zhang W., Li X., Zhang Y., Li L., Chen H. (2025). The relationship of depression, gut microbiota and colorectal cancer: A negative cycle. Well-Being Sci. Rev..

[13] Lynch S.V., Pedersen O. (2016). The human intestinal microbiome in health and disease. N. Engl. J. Med..

[14] Relman D.A. (2012). The human microbiome: Ecosystem resilience and health. Nutr. Rev..

[15] Alhaj O.A., Ibrahim M.O., Elsahoryi N.A., Al-Maseimi O.D. (2026). Processing-Induced Modifications of Camel Milk Immunoglobulins and Lactoferrin: Implications for Immunocompromised Pediatric Populations and Therapeutic Applications. Foods.

[16] Li Y., Ma Q., Li M., Liu W., Liu Y., Wang M., Wang C., Khan M.Z. (2025). Non-bovine milk as functional foods with focus on their antioxidant and anti-inflammatory bioactivities. Antioxidants.

[17] Bielecka M., Cichosz G., Czeczot H. (2022). Antioxidant, antimicrobial and anticarcinogenic activities of bovine milk proteins and their hydrolysates—A review. Int. Dairy J..

[18] Wang R., Yifei F.Y., Weiru R.Y., Sun S.Y., Lei Y.M., Li Y.X., Lu C.X., Zhai J.N., Bai F.R., Ren F. (2025). Roles of probiotics, prebiotics, and postbiotics in B-cell-mediated immune regulation. J. Nutr..

[19] Elsahoryi N.A., Al-Sayyed H.F. (2020). Manufacture of dairy and non-dairy camel milk products. Handbook of Research on Health and Environmental Benefits of Camel Products.

[20] Sánchez C., Franco L., Regal P., Lamas A., Cepeda A., Fente C. (2021). Breast milk: A source of functional compounds with potential application in nutrition and therapy. Nutrients.

[21] Alhaj O., Kanekanian A. (2014). Milk-derived bioactive components from fermentation. Milk and Dairy Products as Functional Foods.

[22] Mishra S.P., Karunakar P., Taraphder S., Yadav H. (2020). Free Fatty Acid Receptors 2 and 3 as Microbial Metabolite Sensors to Shape Host Health: Pharmacophysiological View. Biomedicines.

[23] Parada Venegas D., De la Fuente M.K., Landskron G., González M.J., Quera R., Dijkstra G., Harmsen H.J.M., Faber K.N., Hermoso M.A. (2019). Short Chain Fatty Acids (SCFAs)-Mediated Gut Epithelial and Immune Regulation and Its Relevance for Inflammatory Bowel Diseases. Front. Immunol..

[24] Sankarganesh P., Bhunia A., Kumar A.G., Babu S., Gopukumar S., Lokesh E. (2025). Short-chain fatty acids (SCFAs) in gut health: Implications for drug metabolism and therapeutics. Med. Microecol..

[25] Zhang J., Zhang X., Wu J., Mu G., Wu X. (2026). Dual-Phase Immunomodulation by the Bovine β-Casein Peptide KEMPFPK: Insights into Potential TLR Interaction and Gut Microbiota-Mediated Effects. Foods.

[26] Xu W., Cao F., Zhao M., Fu X., Yin S., Sun Y., Valencak T.G., Ren D. (2022). Macrophage activation by exopolysaccharides from *Streptococcus thermophilus* fermented milk through TLRs-mediated NF-κB and MAPK pathways. Int. Immunopharmacol..

[27] Zhang J., Zhang X., Mu G., Wu X., Wu J. (2025). Bovine β-Casein Peptide YPFPGPIH Regulates Inflammation and Macrophage Activity via TLR/NF-κB/MAPK Signaling. Foods.

[28] Chen H., Sun J., Fang H., Lin Y., Wu H., Lin D., Yang Z., Zhou Q., Zhao B., Zhou T. (2025). Food-derived bioactive peptides: Health benefits, structure–activity relationships, and translational prospects. J. Zhejiang Univ. Sci. B.

[29] Chen C., Xia P., Gan Y., Zheng X., Yang P., Shi A., Liu X., Zhang J., Yu P., Zhang D. (2025). Food-derived bioactive peptides as emerging therapeutic agents: Unlocking novel strategies for colorectal cancer treatment. Pharmacol. Res..

[30] Reyes-Díaz A., González-Córdova A.F., Hernández-Mendoza A., Reyes-Díaz R., Vallejo-Cordoba B. (2018). Immunomodulation by hydrolysates and peptides derived from milk proteins. Int. J. Dairy Technol..

[31] Rashidbeygi E., Samarin M.M., Sheikhhossein F., Khalilkhaneh A.H., Gholizadeh M., Lohrasbi N., Abbasi A., Bazyar H., Askari G., Amini M.R. (2025). The Effect of Kefir Consumption on Blood Pressure and C-Reactive Protein: A Systematic Review and Meta-Analysis of Randomised Controlled Trials. Endocrinol. Diabetes Metab..

[32] Hamsho M., Hawari R., Yesil Z., Dakhel Z., Saydam D.D., Terzi M., Ranneh Y. (2025). Effect of different kefir dosages on inflammation status, metabolic profile, and anthropometric measurements in adults: A systematic review and meta-analysis. Nutr. Metab. Cardiovasc. Dis..

[33] Companys J., Pla-Pagà L., Calderón-Pérez L., Llauradó E., Solà R., Pedret A., Valls R.M. (2020). Fermented dairy products, probiotic supplementation, and cardiometabolic diseases: A systematic review and meta-analysis. Adv. Nutr..

[34] Acevedo-Román A., Pagán-Zayas N., Velázquez-Rivera L.I., Torres-Ventura A.C., Godoy-Vitorino F. (2024). Insights into gut dysbiosis: Inflammatory diseases, obesity, and restoration approaches. Int. J. Mol. Sci..

[35] Abeltino A., Hatem D., Serantoni C., Riente A., De Giulio M.M., De Spirito M., De Maio F., Maulucci G. (2024). Unraveling the gut microbiota: Implications for precision nutrition and personalized medicine. Nutrients.

[36] Hess J.M., Jonnalagadda S.S., Slavin J.L. (2016). Dairy foods: Current evidence of their effects on bone, cardiometabolic, cognitive, and digestive health. Compr. Rev. Food Sci. Food Saf..

[37] Thorman A.W., Adkins G., Conrey S.C., Burrell A.R., Yu Y., White B., Burke R., Haslam D., Payne D.C., Staat M.A. (2023). Gut Microbiome Composition and Metabolic Capacity Differ by *FUT2* Secretor Status in Exclusively Breastfed Infants. Nutrients.

[38] Jardon K.M., Canfora E.E., Goossens G.H., Blaak E.E. (2022). Dietary macronutrients and the gut microbiome: A precision nutrition approach to improve cardiometabolic health. Gut.

[39] Thoniyot S., Balakrishnan V. (2025). Integrating multi-omics data for personalized nutrition using knowledge graphs and Graph Neural Networks: A Comprehensive Review. Comput. Biol. Chem..

[40] Donovan S.M. (2019). Human milk proteins: Composition and physiological significance. Proceedings of the Nestle Nutrition Institute Workshop Series.

[41] Alhaj O.A. (2020). Exploring potential therapeutic properties of camel milk. Handbook of Research on Health and Environmental Benefits of Camel Products.

[42] García-Montoya I.A., Cendón T.S., Arévalo-Gallegos S., Rascón-Cruz Q. (2012). Lactoferrin a multiple bioactive protein: An overview. Biochim. Biophys. Acta.

[43] Cao X., Ren Y., Lu Q., Wang K., Wu Y., Wang Y., Zhang Y., Cui X.S., Yang Z., Chen Z. (2022). Lactoferrin: A glycoprotein that plays an active role in human health. Front. Nutr..

[44] Vega-Bautista A., de la Garza M., Carrero J.C., Campos-Rodríguez R., Godínez-Victoria M., Drago-Serrano M.E. (2019). The Impact of Lactoferrin on the Growth of Intestinal Inhabitant Bacteria. Int. J. Mol. Sci..

[45] Zhao C., Chen N., Ashaolu T.J. (2023). Prebiotic and modulatory evidence of lactoferrin on gut health and function. J. Funct. Foods.

[46] Giansanti F., Panella G., Leboffe L., Antonini G. (2016). Lactoferrin from milk: Nutraceutical and pharmacological properties. Pharmaceuticals.

[47] Permyakov E.A. (2020). α-Lactalbumin, amazing calcium-binding protein. Biomolecules.

[48] Permyakov E.A., Berliner L.J. (2000). α-Lactalbumin: Structure and function. FEBS Lett..

[49] Eker F., Akdaşçi E., Duman H., Yalçıntaş Y.M., Canbolat A.A., Kalkan A.E., Karav S., Šamec D. (2024). Antimicrobial Properties of Colostrum and Milk. Antibiotics.

[50] Montagne P., Cuillière M.L., Molé C., Béné M.C., Faure G. (2001). Changes in lactoferrin and lysozyme levels in human milk during the first twelve weeks of lactation. Adv. Exp. Med. Biol..

[51] Neelima, Sharma R., Rajput Y.S., Mann B. (2013). Chemical and functional properties of glycomacropeptide (GMP) and its role in the detection of cheese whey adulteration in milk: A review. Dairy Sci. Technol..

[52] Córdova-Dávalos L.E., Jiménez M., Salinas E. (2019). Glycomacropeptide bioactivity and health: A review highlighting action mechanisms and signaling pathways. Nutrients.

[53] O’Riordan N., O’Callaghan J., Buttò L.F., Kilcoyne M., Joshi L., Hickey R.M. (2018). Bovine glycomacropeptide promotes the growth of *Bifidobacterium longum* ssp. *infantis* and modulates its gene expression. J. Dairy Sci..

[54] Zhu L., Li H., Luo T., Deng Z., Li J., Zheng L., Zhang B. (2023). Human Milk Oligosaccharides: A Critical Review on Structure, Preparation, Their Potential as a Food Bioactive Component, and Future Perspectives. J. Agric. Food Chem..

[55] Spicer S.K., Gaddy J.A., Townsend S.D. (2022). Recent advances on human milk oligosaccharide antimicrobial activity. Curr. Opin. Chem. Biol..

[56] Zheng J., Xu H., Fang J., Zhang X. (2022). Enzymatic and chemoenzymatic synthesis of human milk oligosaccharides and derivatives. Carbohydr. Polym..

[57] Gopal P.K., Gill H. (2000). Oligosaccharides and glycoconjugates in bovine milk and colostrum. Br. J. Nutr..

[58] Bode L. (2012). Human milk oligosaccharides: Every baby needs a sugar mama. Glycobiology.

[59] Isernhagen L., Galuska C.E., Vernunft A., Galuska S.P. (2024). Structural Characterization and Abundance of Sialylated Milk Oligosaccharides in Holstein Cows during Early Lactation. Foods.

[60] Soyyılmaz B., Mikš M.H., Röhrig C.H., Matwiejuk M., Meszaros-Matwiejuk A., Vigsnæs L.K. (2021). The Mean of Milk: A Review of Human Milk Oligosaccharide Concentrations throughout Lactation. Nutrients.

[61] Kenney A.D., Sabag-Daigle A., Stoecklein M.-M., Buck R.H., Reverri E.J. (2025). A review of human milk oligosaccharide concentrations of breast milk for infants and young children through 24 months of age. Front. Pediatr..

[62] Nogacka A.M., Cuesta I., Gueimonde M., de los Reyes-Gavilán C.G. (2023). 2-Fucosyllactose Metabolism by Bifidobacteria Promotes Lactobacilli Growth in Co-Culture. Microorganisms.

[63] Thongaram T., Hoeflinger J.L., Chow J., Miller M.J. (2017). Human milk oligosaccharide consumption by probiotic and human-associated bifidobacteria and lactobacilli. J. Dairy Sci..

[64] Musilova S., Rada V., Vlkova E., Bunesova V. (2014). Beneficial effects of human milk oligosaccharides on gut microbiota. Benef. Microbes.

[65] He Y., Liu S., Kling D.E., Leone S., Lawlor N.T., Huang Y., Feinberg S.B., Hill D.R., Newburg D.S. (2016). The human milk oligosaccharide 2′-fucosyllactose modulates CD14 expression in human enterocytes, thereby attenuating LPS-induced inflammation. Gut.

[66] Wang Y., Zou Y., Wang J., Ma H., Zhang B., Wang S. (2020). The Protective Effects of 2′-Fucosyllactose against *E. Coli* O157 Infection Are Mediated by the Regulation of Gut Microbiota and the Inhibition of Pathogen Adhesion. Nutrients.

[67] Ruiz-Palacios G., Cervantes L., Ramos P., Chavez-Munguia B., Newburg D. (2003). Campylobacter jejuni binds intestinal H(O) antigen and fucosyloligosaccharides of human milk inhibit its binding and infection. J. Biol. Chem..

[68] Ehrlich A.M., Pacheco A.R., Henrick B.M., Taft D., Xu G., Huda M.N., Mishchuk D., Goodson M.L., Slupsky C., Barile D. (2020). Indole-3-lactic acid associated with Bifidobacterium-dominated microbiota significantly decreases inflammation in intestinal epithelial cells. BMC Microbiol..

[69] Kezer G., Paramithiotis S., Khwaldia K., Harahap I.A., Čagalj M., Šimat V., Smaoui S., Elfalleh W., Ozogul F., Esatbeyoglu T. (2025). A comprehensive overview of the effects of probiotics, prebiotics and synbiotics on the gut-brain axis. Front. Microbiol..

[70] Zhai J., Li Y., Liu J., Dai C. (2026). Gut microbiota and central nervous system’s direct bidirectional regulation: The mechanisms of the gut–brain axis in irritable bowel syndrome. Clin. Transl. Discov..

[71] Meleti E., Koureas M., Manouras A., Giannouli P., Malissiova E. (2025). Bioactive Peptides from Dairy Products: A Systematic Review of Advances, Mechanisms, Benefits, and Functional Potential. Dairy.

[72] Bechaux J., Gatellier P., Le Page J., Drillet Y., Sante-Lhoutellier V. (2019). A comprehensive review on bioactive peptides from animal by-products and their applications. Food Funct..

[73] Ye H., Tao X., Zhang W., Chen Y., Yu Q., Xie J. (2022). Food-derived bioactive peptides: Production, biological activities, opportunities and challenges. J. Future Foods.

[74] Bütikofer U., Meyer J., Sieber R., Walther B., Wechsler D. (2008). Occurrence of the angiotensin-converting enzyme–inhibiting tripeptides Val-Pro-Pro and Ile-Pro-Pro in different cheese varieties of Swiss origin. J. Dairy Sci..

[75] Ohsawa K., Satsu H., Ohki K., Enjoh M., Takano T., Shimizu M. (2008). Producibility and digestibility of antihypertensive β-casein tripeptides, Val-Pro-Pro and Ile-Pro-Pro, in the gastrointestinal tract: Analyses using an in vitro model of mammalian gastrointestinal digestion. J. Agric. Food Chem..

[76] Alhaj O.A., Abodoleh G., Abu Jadayil S., Irshaid N., Mehta B.M., Faye B., Jahrami H.A. (2024). A systematic review and meta-analysis of the antihypertensive effects of camel milk hydrolysates. Int. J. Dairy Technol..

[77] Alhaj O.A., Jrad Z., Oussaief O., Jahrami H.A., Ahmad L., Alshuniaber M.A., Mehta B.M. (2024). The characterization of *Lactobacillus* strains in camel and bovine milk during fermentation: A comparison study. Heliyon.

[78] Gao X., Zhao J., Zhang H., Chen W., Zhai Q. (2022). Modulation of gut health using probiotics: The role of probiotic effector molecules. J. Future Foods.

[79] Xu C., Yan S., Guo Y., Qiao L., Ma L., Dou X., Zhang B. (2020). *Lactobacillus casei* ATCC 393 alleviates Enterotoxigenic *Escherichia coli* K88-induced intestinal barrier dysfunction via TLRs/mast cells pathway. Life Sci..

[80] Chen Y., You Y., Ren L., Fu G., Zhou N., Xiao Y., Shi D. (2025). Exploration of a Postbiotic Derived from *Enterococcus faecium* HDRsEf1 and Its Probiotic Mechanisms. Microorganisms.

[81] Wijesekara T., Abeyrathne E.D.N.S., Ahn D.U. (2024). Effect of Bioactive Peptides on Gut Microbiota and Their Relations to Human Health. Foods.

[82] Drago-Serrano M.E., de la Garza-Amaya M., Luna J.S., Campos-Rodríguez R. (2012). Lactoferrin-lipopolysaccharide (LPS) binding as key to antibacterial and antiendotoxic effects. Int. Immunopharmacol..

[83] Xie D., Shen Y., Su E., Du L., Xie J., Wei D. (2023). Anti-hyperuricemic, nephroprotective, and gut microbiota regulative effects of separated hydrolysate of α-lactalbumin on potassium oxonate-and hypoxanthine-induced hyperuricemic mice. Mol. Nutr. Food Res..

[84] Baron F., Nau F., Guérin-Dubiard C., Bonnassie S., Gautier M., Andrews S.C., Jan S. (2016). Egg white versus Salmonella Enteritidis! A harsh medium meets a resilient pathogen. Food Microbiol..

[85] Khorshidian N., Khanniri E., Koushki M.R., Sohrabvandi S., Yousefi M. (2022). An Overview of Antimicrobial Activity of Lysozyme and Its Functionality in Cheese. Front. Nutr..

[86] Rada V., Splichal I., Rockova S., Grmanova M., Vlkova E. (2010). Susceptibility of bifidobacteria to lysozyme as a possible selection criterion for probiotic bifidobacterial strains. Biotechnol. Lett..

[87] Sawin E.A., De Wolfe T.J., Aktas B., Stroup B.M., Murali S.G., Steele J.L., Ney D.M. (2015). Glycomacropeptide is a prebiotic that reduces *Desulfovibrio* bacteria, increases cecal short-chain fatty acids, and is anti-inflammatory in mice. Am. J. Physiol.-Gastrointest. Liver Physiol..

[88] Li Y., Yang S., Lun J., Gao J., Gao X., Gong Z., Wan Y., He X., Cao H. (2020). Inhibitory Effects of the *Lactobacillus rhamnosus* GG Effector Protein HM0539 on Inflammatory Response Through the TLR4/MyD88/NF-κB Axis. Front. Immunol..

[89] Leser T., Baker A. (2024). Molecular Mechanisms of *Lacticaseibacillus rhamnosus*, LGG^®^ Probiotic Function. Microorganisms.

[90] Yan F., Cao H., Cover T.L., Whitehead R., Washington M.K., Polk D.B. (2007). Soluble proteins produced by probiotic bacteria regulate intestinal epithelial cell survival and growth. Gastroenterology.

[91] Ismael M., Huang M., Zhong Q. (2024). The Bacteriocins Produced by Lactic Acid Bacteria and the Promising Applications in Promoting Gastrointestinal Health. Foods.

[92] Darbandi A., Asadi A., Mahdizade Ari M., Ohadi E., Talebi M., Halaj Zadeh M., Darb Emamie A., Ghanavati R., Kakanj M. (2022). Bacteriocins: Properties and potential use as antimicrobials. J. Clin. Lab. Anal..

[93] Ward R.E., Niñonuevo M., Mills D.A., Lebrilla C.B., German J.B. (2006). In vitro fermentation of breast milk oligosaccharides by *Bifidobacterium infantis* and *Lactobacillus gasseri*. Appl. Environ. Microbiol..

[94] Wakinaka T., Kiyohara M., Kurihara S., Hirata A., Chaiwangsri T., Ohnuma T., Fukamizo T., Katayama T., Ashida H., Yamamoto K. (2013). Bifidobacterial α-galactosidase with unique carbohydrate-binding module specifically acts on blood group B antigen. Glycobiology.

[95] Thomson P., Medina D.A., Garrido D. (2018). Human milk oligosaccharides and infant gut bifidobacteria: Molecular strategies for their utilization. Food Microbiol..

[96] Vandenplas Y., Berger B., Carnielli V.P., Ksiazyk J., Lagström H., Sanchez Luna M., Migacheva N., Mosselmans J.M., Picaud J.C., Possner M. (2018). Human Milk Oligosaccharides: 2′-Fucosyllactose (2′-FL) and Lacto-N-Neotetraose (LNnT) in Infant Formula. Nutrients.

[97] Zhang T., Chen G., Zeng J., Xiong G., Du C., Sun Y., Zeng X., Chen C. (2025). Sialylated IgG mediates the colonization of *Bifidobacterium bifidum* through the “FcRn-Sialylated IgG-SiaBb2” axis, alleviating high-fructose diet-induced neuroinflammation in mice by activating the cAMP/PKA signaling pathway. Food Biosci..

[98] Miller-Catchpole R., Kot E., Haloftis G., Furmanov S., Bezkorovainy A. (1997). Lactoferrin can supply iron for the growth of *Bifidobacterium breve*. Nutr. Res..

[99] Woodman T., Strunk T., Patole S., Hartmann B., Simmer K., Currie A. (2018). Effects of lactoferrin on neonatal pathogens and *Bifidobacterium breve* in human breast milk. PLoS ONE.

[100] Liu Z.-S., Lin C.-F., Chen P.-W. (2021). Transcriptome analysis of *Lactobacillus rhamnosus* GG strain treated with prebiotic-bovine lactoferrin under a cold environment. J. Food Drug Anal..

[101] Morozumi M., Wada Y., Tsuda M., Tabata F., Ehara T., Nakamura H., Miyaji K. (2023). Cross-feeding among bifidobacteria on glycomacropeptide. J. Funct. Foods.

[102] Ashaolu T.J., Lee C.C., Tarhan O., Rashidinejad A., Jafari S.M. (2026). Nexus of Whey Proteins, Gut Dysbiosis, and Colonic Health. Food Sci. Nutr..

[103] Facchin S., Bertin L., Bonazzi E., Lorenzon G., De Barba C., Barberio B., Zingone F., Maniero D., Scarpa M., Ruffolo C. (2024). Short-chain fatty acids and human health: From metabolic pathways to current therapeutic implications. Life.

[104] Calatayud M., Börner R.A., Ghyselinck J., Verstrepen L., Medts J.D., Abbeele P.V.d., Boulangé C.L., Priour S., Marzorati M., Damak S. (2021). Water kefir and derived pasteurized beverages modulate gut microbiota, intestinal permeability and cytokine production in vitro. Nutrients.

[105] Ma L., Tu H., Chen T. (2023). Postbiotics in human health: A narrative review. Nutrients.

[106] Rose E.C., Odle J., Blikslager A.T., Ziegler A.L. (2021). Probiotics, prebiotics and epithelial tight junctions: A promising approach to modulate intestinal barrier function. Int. J. Mol. Sci..

[107] Kobayashi M., Mikami D., Kimura H., Kamiyama K., Morikawa Y., Yokoi S., Kasuno K., Takahashi N., Taniguchi T., Iwano M. (2017). Short-chain fatty acids, GPR41 and GPR43 ligands, inhibit TNF-α-induced MCP-1 expression by modulating p38 and JNK signaling pathways in human renal cortical epithelial cells. Biochem. Biophys. Res. Commun..

[108] Chen H., Xu Y., Qiu J., Guan Z., Yu S., Ding Y. (2026). Differential regulatory mechanisms of gut-derived SCFAs (acetate, propionate, butyrate) on the pulmonary TLR2 signaling pathway and their roles in the inflammatory balance of bacterial pneumonia. Eur. J. Med. Res..

[109] Porbahaie M., Hummel A., Saouadogo H., Coelho R.M.L., Savelkoul H.F.J., Teodorowicz M., van Neerven R.J.J. (2023). Short-chain fatty acids inhibit the activation of T lymphocytes and myeloid cells and induce innate immune tolerance. Benef. Microbes.

[110] Veiga P., Pons N., Agrawal A., Oozeer R., Guyonnet D., Brazeilles R., Faurie J.M., van Hylckama Vlieg J.E., Houghton L.A., Whorwell P.J. (2014). Changes of the human gut microbiome induced by a fermented milk product. Sci. Rep..

[111] Monteiro C., Bogea E.G., Campos C.D.L., Pereira-Filho J.L., Almeida V.S.S., Vale A.A.M., Azevedo-Santos A.P.S., Monteiro-Neto V. (2026). Prebiotics and Gut Health: Mechanisms, Clinical Evidence, and Future Directions. Nutrients.

[112] Xu Z., Wang T., Wang Y., Li Y., Sun Y., Qiu H.-J. (2025). Short-chain fatty acids: Key antiviral mediators of gut microbiota. Front. Immunol..

[113] Callaway T., Edrington T., Anderson R., Harvey R., Genovese K., Kennedy C., Venn D., Nisbet D. (2008). Probiotics, prebiotics and competitive exclusion for prophylaxis against bacterial disease. Anim. Health Res. Rev..

[114] Kosznik-Kwaśnicka K., Leszczyńska U., Piechowicz L. (2026). Lactoferrin bridges antimicrobial and healing responses in *Staphylococcus aureus* skin infections. Front. Microbiol..

[115] Ohradanova-Repic A., Praženicová R., Gebetsberger L., Moskalets T., Skrabana R., Cehlar O., Tajti G., Stockinger H., Leksa V. (2023). Time to kill and time to heal: The multifaceted role of lactoferrin and lactoferricin in host defense. Pharmaceutics.

[116] Hu P., Zhao F., Wang J., Zhu W. (2020). Lactoferrin attenuates lipopolysaccharide-stimulated inflammatory responses and barrier impairment through the modulation of NF-κB/MAPK/Nrf2 pathways in IPEC-J2 cells. Food Funct..

[117] Park C., Cha H.-J., Lee H., Kim G.-Y., Choi Y.H. (2021). The regulation of the TLR4/NF-κB and Nrf2/HO-1 signaling pathways is involved in the inhibition of lipopolysaccharide-induced inflammation and oxidative reactions by morroniside in RAW 264.7 macrophages. Arch. Biochem. Biophys..

[118] Bruni N., Capucchio M.T., Biasibetti E., Pessione E., Cirrincione S., Giraudo L., Corona A., Dosio F. (2016). Antimicrobial Activity of Lactoferrin-Related Peptides and Applications in Human and Veterinary Medicine. Molecules.

[119] Pei J., Xiong L., Wu X., Chu M., Bao P., Ge Q., Guo X. (2025). Bovine lactoferricin exerts antibacterial activity against four Gram-negative pathogenic bacteria by transforming its molecular structure. Front. Cell. Infect. Microbiol..

[120] Nawaz N., Wen S., Wang F., Nawaz S., Raza J., Iftikhar M., Usman M. (2022). Lysozyme and Its Application as Antibacterial Agent in Food Industry. Molecules.

[121] Sakurai T., Hashikura N., Minami J., Yamada A., Odamaki T., Xiao J.-Z. (2017). Tolerance mechanisms of human-residential bifidobacteria against lysozyme. Anaerobe.

[122] Ma B., Gavzy S.J., Saxena V., Song Y., Piao W., Lwin H.W., Lakhan R., Iyyathurai J., Li L., France M. (2023). Strain-specific alterations in gut microbiome and host immune responses elicited by tolerogenic Bifidobacterium pseudolongum. Sci. Rep..

[123] Rodriguez E., Arques J.L., Nunez M., Gaya P., Medina M. (2005). Combined effect of high-pressure treatments and bacteriocin-producing lactic acid bacteria on inactivation of *Escherichia coli* O157: H7 in raw-milk cheese. Appl. Environ. Microbiol..

[124] Al-Holy M.A., Al-Nabulsi A., Osaili T.M., Ayyash M.M., Shaker R.R. (2012). Inactivation of Listeria innocua in brined white cheese by a combination of nisin and heat. Food Control.

[125] Schlesser J.E., Gerdes R., Ravishankar S., Madsen K., Mowbray J., Teo A.Y. (2006). Survival of a five-strain cocktail of *Escherichia coli* O157:H7 during the 60-day aging period of cheddar cheese made from unpasteurized milk. J. Food Prot..

[126] Castellani C., Obermüller B., Kienesberger B., Singer G., Peterbauer C., Grabherr R., Mayrhofer S., Klymiuk I., Horvath A., Stadlbauer V. (2021). Production, storage stability, and susceptibility testing of reuterin and its impact on the murine fecal microbiome and volatile organic compound profile. Front. Microbiol..

[127] Iqbal Z., Ahmed S., Tabassum N., Bhattacharya R., Bose D. (2021). Role of probiotics in prevention and treatment of enteric infections: A comprehensive review. 3 Biotech.

[128] Buddhasiri S., Sukjoi C., Kaewsakhorn T., Nambunmee K., Nakphaichit M., Nitisinprasert S., Thiennimitr P. (2021). Anti-inflammatory effect of probiotic *Limosilactobacillus reuteri* KUB-AC5 against *Salmonella* infection in a mouse colitis model. Front. Microbiol..

[129] Pražnikar Z.J., Kenig S., Vardjan T., Bizjak M.Č., Petelin A. (2020). Effects of kefir or milk supplementation on zonulin in overweight subjects. J. Dairy Sci..

[130] Mataigne A., Vannier N., Vandenkoornhuyse P., Hacquard S. (2021). Microbial Systems Ecology to Understand Cross-Feeding in Microbiomes. Front. Microbiol..

[131] Moens F., Verce M., De Vuyst L. (2017). Lactate- and acetate-based cross-feeding interactions between selected strains of lactobacilli, bifidobacteria and colon bacteria in the presence of inulin-type fructans. Int. J. Food Microbiol..

[132] Shinohara R., Sasaki K., Inoue J., Hoshi N., Fukuda I., Sasaki D., Kondo A., Osawa R. (2019). Butyryl-CoA:acetate CoA-transferase gene associated with the genus *Roseburia* is decreased in the gut microbiota of Japanese patients with ulcerative colitis. Biosci. Microbiota Food Health.

[133] Tejedor-Sanz S., Stevens E.T., Li S., Finnegan P., Nelson J., Knoesen A., Light S.H., Ajo-Franklin C.M., Marco M.L. (2022). Extracellular electron transfer increases fermentation in lactic acid bacteria via a hybrid metabolism. Elife.

[134] Li Q., Marietou A., Andersen F.F., Hosek J., Scavenius C., Zhang J., Schwab C. (2025). In vitro investigations on the impact of fermented dairy constituents on fecal microbiota composition and fermentation activity. Microbiol. Spectr..

[135] Öneş E., Zavotçu M., Nisan N., Baş M., Sağlam D. (2025). Effects of Kefir Consumption on Gut Microbiota and Athletic Performance in Professional Female Soccer Players: A Randomized Controlled Trial. Nutrients.

[136] Nikniaz Z., Tabrizi J.S., Ghojazadeh M., Farhangi M.A., Hosseini M.S., Allameh M., Norouzi S., Nikniaz L. (2020). Community-based interventions to increase dairy intake in healthy populations: A systematic review. Public Health Rev..

[137] Bellikci-Koyu E., Sarer-Yurekli B.P., Akyon Y., Aydin-Kose F., Karagozlu C., Ozgen A.G., Brinkmann A., Nitsche A., Ergunay K., Yilmaz E. (2019). Effects of Regular Kefir Consumption on Gut Microbiota in Patients with Metabolic Syndrome: A Parallel-Group, Randomized, Controlled Study. Nutrients.

[138] Sadiq F.A., De Reu K., Yang N., Burmølle M., Heyndrickx M. (2024). Interspecies interactions in dairy biofilms drive community structure and response against cleaning and disinfection. Biofilm.

[139] Zhang Q., Ren J., Zhao H., Zhao M., Xu J., Zhao Q. (2011). Influence of casein hydrolysates on the growth and lactic acid production of *Lactobacillus delbrueckii* subsp. *bulgaricus* and *Streptococcus thermophilus*. Int. J. Food Sci. Technol..

[140] Settachaimongkon S., Nout M.R., Fernandes E.C.A., Hettinga K.A., Vervoort J.M., van Hooijdonk T.C., Zwietering M.H., Smid E.J., van Valenberg H.J. (2014). Influence of different proteolytic strains of *Streptococcus thermophilus* in co-culture with *Lactobacillus delbrueckii* subsp. *bulgaricus* on the metabolite profile of set-yoghurt. Int. J. Food Microbiol..

[141] Herve-Jimenez L., Guillouard I., Guedon E., Boudebbouze S., Hols P., Monnet V., Maguin E., Rul F. (2009). Postgenomic analysis of *Streptococcus thermophilus* cocultivated in milk with *Lactobacillus delbrueckii* subsp. *bulgaricus*: Involvement of nitrogen, purine, and iron metabolism. Appl. Environ. Microbiol..

[142] Wachamo S., Gaultier A. (2025). The emerging role of microbiota derived SCFAs in neurodegenerative disorders. Brain Behav. Immun.-Health.

[143] Geirnaert A., Calatayud M., Grootaert C., Laukens D., Devriese S., Smagghe G., De Vos M., Boon N., Van de Wiele T. (2017). Butyrate-producing bacteria supplemented in vitro to Crohn’s disease patient microbiota increased butyrate production and enhanced intestinal epithelial barrier integrity. Sci. Rep..

[144] Hodgkinson K., El Abbar F., Dobranowski P., Manoogian J., Butcher J., Figeys D., Mack D., Stintzi A. (2023). Butyrate’s role in human health and the current progress towards its clinical application to treat gastrointestinal disease. Clin. Nutr..

[145] Barbara G., Barbaro M.R., Fuschi D., Palombo M., Falangone F., Cremon C., Marasco G., Stanghellini V. (2021). Inflammatory and Microbiota-Related Regulation of the Intestinal Epithelial Barrier. Front. Nutr..

[146] Vancamelbeke M., Vermeire S. (2017). The intestinal barrier: A fundamental role in health and disease. Expert Rev. Gastroenterol. Hepatol..

[147] Lee B., Moon K.M., Kim C.Y. (2018). Tight Junction in the Intestinal Epithelium: Its Association with Diseases and Regulation by Phytochemicals. J. Immunol. Res..

[148] Monaco A., Ovryn B., Axis J., Amsler K. (2021). The Epithelial Cell Leak Pathway. Int. J. Mol. Sci..

[149] Guzmán-Mejía F., Molotla-Torres D.E., Godínez-Victoria M., Valdes-Hilarios X., Sánchez-Miranda E., Oros-Pantoja R., Drago-Serrano M.E. (2024). Looking Inside of the Intestinal Permeability Regulation by Protein-Derivatives from Bovine Milk. Mol. Nutr. Food Res..

[150] Ulluwishewa D., Mullaney J., Adam K., Claycomb R., Anderson R.C. (2022). A bioactive bovine whey protein extract improves intestinal barrier function in vitro. JDS Commun..

[151] Seth A., Yan F., Polk D.B., Rao R.K. (2008). Probiotics ameliorate the hydrogen peroxide-induced epithelial barrier disruption by a PKC- and MAP kinase-dependent mechanism. Am. J. Physiol. Gastrointest. Liver Physiol..

[152] Guo H., Yu L., Tian F., Chen W., Zhai Q. (2023). The potential therapeutic role of *Lactobacillaceae rhamnosus* for treatment of inflammatory bowel disease. Foods.

[153] Gao J., Li Y., Wan Y., Hu T., Liu L., Yang S., Gong Z., Zeng Q., Wei Y., Yang W. (2019). A novel postbiotic from *Lactobacillus rhamnosus* GG with a beneficial effect on intestinal barrier function. Front. Microbiol..

[154] Yang S., Xu X., Peng Q., Ma L., Qiao Y., Shi B. (2023). Exopolysaccharides from lactic acid bacteria, as an alternative to antibiotics, on regulation of intestinal health and the immune system. Anim. Nutr..

[155] Illikoud N., Mantel M., Rolli-Derkinderen M., Gagnaire V., Jan G. (2022). Dairy starters and fermented dairy products modulate gut mucosal immunity. Immunol. Lett..

[156] Lao L., Yang G., Zhang A., Liu L., Guo Y., Lian L., Pan D., Wu Z. (2022). Anti-inflammation and gut microbiota regulation properties of fatty acids derived from fermented milk in mice with dextran sulfate sodium-induced colitis. J. Dairy Sci..

[157] Yu W., Sun S., Yan Y., Zhou H., Liu Z., Fu Q. (2025). The role of short-chain fatty acid in metabolic syndrome and its complications: Focusing on immunity and inflammation. Front. Immunol..

[158] Le Do Q., Malau I.A., Nguyen H.T., Liu J., Chang J.P.-C., Su K.-P. (2026). Circulating Short-Chain Fatty Acid (SCFA) Profiles as a Biomarker of Gut-Brain Axis Dysfunction: A Meta-Analysis for the SCFA Signature in Major Depression. Biomed. J..

[159] Modrego J., Pantoja-Arévalo L., Gómez-Garre D., Gesteiro E., González-Gross M. (2025). Dairy-Gut Microbiome Interactions: Implications for Immunity, Adverse Reactions to Food, Physical Performance and Cardiometabolic Health—A Narrative Review. Nutrients.

[160] Yuan F., Han X., Huang M., Su Y., Zhang Y., Hu M., Yu X., Jin W., Li Y., Zhang L. (2025). The human milk-derived peptide drives rapid regulation of macrophage inflammation responses in the neonatal intestine. Cell. Mol. Gastroenterol. Hepatol..

[161] Lee G., Jin Y., Lee S.A., Lee S.-Y., Lee H., Nan Z., Yoon C.-S., Lee D.-S. (2026). Anti-Neuroinflammatory Effects of a Representative Low-Molecular-Weight Component Isolated from *Codium fragile* Through Inhibition of the NF-κB Pathway in Microglia and Macrophage Cells. Mar. Drugs.

[162] Shao J., Li Y., Wang Z., Xiao M., Yin P., Lu Y., Qian X., Xu Y., Liu J. (2013). 7b, a novel naphthalimide derivative, exhibited anti-inflammatory effects via targeted-inhibiting TAK1 following down-regulation of ERK1/2-and p38 MAPK-mediated activation of NF-κB in LPS-stimulated RAW264. 7 macrophages. Int. Immunopharmacol..

[163] Hsia C.-H., Velusamy M., Jayakumar T., Chen Y.-J., Hsia C.-W., Tsai J.-H., Teng R.-D., Sheu J.-R. (2018). Mechanisms of TQ-6, a novel ruthenium-derivative compound, against lipopolysaccharide-induced in vitro macrophage activation and liver injury in experimental mice: The crucial role of p38 MAPK and NF-κB signaling. Cells.

[164] Panahipour L., Kochergina E., Kreissl A., Haiden N., Gruber R. (2019). Milk modulates macrophage polarization in vitro. Cytokine X.

[165] Cai J., Yan X., Liu X., Yin X., Shi A., Ji C., Cao Y. (2025). Human β-casein-derived peptide BCCY-1 improved the intestinal barrier integrity by regulating the TLR4/eNOS/3-Nitrotyrosine axis. Food Chem..

[166] Henrick B.M., Rodriguez L., Lakshmikanth T., Pou C., Henckel E., Arzoomand A., Olin A., Wang J., Mikes J., Tan Z. (2021). Bifidobacteria-mediated immune system imprinting early in life. Cell.

[167] Yao K., Zeng L., He Q., Wang W., Lei J., Zou X. (2017). Effect of probiotics on glucose and lipid metabolism in type 2 diabetes mellitus: A meta-analysis of 12 randomized controlled trials. Med. Sci. Monit. Int. Med. J. Exp. Clin. Res..

[168] Mukarromah T.A., Rustanti N., Mahati E., Suparmi S., Ayustaningwarno F. (2025). The Impact of Fermented Milk Products on Gut Microbiota-Derived Metabolites in Obesity: A Narrative Review. J. Food Sci..

[169] Kibbie J.J., Dillon S.M., Thompson T.A., Purba C.M., McCarter M.D., Wilson C.C. (2021). Butyrate directly decreases human gut lamina propria CD4 T cell function through histone deacetylase (HDAC) inhibition and GPR43 signaling. Immunobiology.

[170] Steliou K., Boosalis M.S., Perrine S.P., Sangerman J., Faller D.V. (2012). Butyrate histone deacetylase inhibitors. Biores. Open Access.

[171] Peng L., Li Z.R., Green R.S., Holzman I.R., Lin J. (2009). Butyrate enhances the intestinal barrier by facilitating tight junction assembly via activation of AMP-activated protein kinase in Caco-2 cell monolayers. J. Nutr..

[172] Szajnar K., Pawlos M., Kowalczyk M., Drobniak J., Znamirowska-Piotrowska A. (2025). Fermented Milk Supplemented with Sodium Butyrate and Inulin: Physicochemical Characterization and Probiotic Viability Under In Vitro Simulated Gastrointestinal Digestion. Nutrients.

[173] Xu S., Boylston T.D., Glatz B.A. (2005). Conjugated linoleic acid content and organoleptic attributes of fermented milk products produced with probiotic bacteria. J. Agric. Food Chem..

[174] Reihnér E., Rudling M., Ståhlberg D., Berglund L., Ewerth S., Björkhem I., Einarsson K., Angelin B. (1990). Influence of pravastatin, a specific inhibitor of HMG-CoA reductase, on hepatic metabolism of cholesterol. N. Engl. J. Med..

[175] Kim D.H., Kim H., Jeong D., Kang I.B., Chon J.W., Kim H.S., Song K.Y., Seo K.H. (2017). Kefir alleviates obesity and hepatic steatosis in high-fat diet-fed mice by modulation of gut microbiota and mycobiota: Targeted and untargeted community analysis with correlation of biomarkers. J. Nutr. Biochem..

[176] Lahrairi M., Jmaili K., Mahmoudi M., Bahlaouan B., Bessi H., Boutaleb N. (2026). The Science of Kefir: Advances in Microbial Ecology, Bioprocess Control, and Functional Health Outcomes. Nat. Built Soc. Environ. Health.

[177] Qaisrani Z.N., Lin W.P., Lay B.B., Phyo K.Y., San M.M., Awaeloh N., Aunsorn S., Pattanayaiying R., Na Ayudthaya S.P., Hongkulsup C. (2025). The impact of kefir consumption on inflammation, oxidative stress status, and metabolic-syndrome-related parameters in animal models: A systematic review and meta-analysis. Foods.

[178] Ejtahed H.S., Mohtadi-Nia J., Homayouni-Rad A., Niafar M., Asghari-Jafarabadi M., Mofid V., Akbarian-Moghari A. (2011). Effect of probiotic yogurt containing *Lactobacillus acidophilus* and *Bifidobacterium lactis* on lipid profile in individuals with type 2 diabetes mellitus. J. Dairy Sci..

[179] da Silva Ghizi A.C., de Almeida Silva M., de Andrade Moraes F.S., da Silva C.L., Endringer D.C., Scherer R., Lenz D., de Lima E.M., Brasil G.A., Maia J.F. (2021). Kefir improves blood parameters and reduces cardiovascular risks in patients with metabolic syndrome. PharmaNutrition.

[180] Yen C.C., Tsai C.L., Chang G.R., Ko C.H., Tu M.Y., Lan Y.W., Chen H.L., Chen C.M. (2025). Kefir-derived exopolysaccharide ameliorates hyperglycemic control and beta cell integrity in a rat model of type 2 diabetes mellitus. Nutr. Diabetes.

[181] Apalowo O.E., Adegoye G.A., Mbogori T., Kandiah J., Obuotor T.M. (2024). Nutritional characteristics, health impact, and applications of kefir. Foods.

[182] Shuai M., Miao Z., Gou W., Xu F., Jiang Z., Ling C.-w., Fu Y., Xiong F., Chen Y.-m., Zheng J.-S. (2021). Multi-omics analyses reveal relationships among dairy consumption, gut microbiota and cardiometabolic health. EBioMedicine.

[183] Mohammad I., Ansari M.R., Khan M.S., Bari M.N., Kamal M.A., Poyil M.M. (2025). Beyond Digestion: The Gut Microbiota as an Immune–Metabolic Interface in Disease Modulation. Gastrointest. Disord..

[184] Robinson S.R., Greenway F.L., Deth R.C., Fayet-Moore F. (2025). Effects of Different Cow-Milk Beta-Caseins on the Gut-Brain Axis: A Narrative Review of Preclinical, Animal, and Human Studies. Nutr. Rev..

[185] Park Y.W., Nam M.S. (2015). Bioactive Peptides in Milk and Dairy Products: A Review. Korean J. Food Sci. Anim. Resour..

[186] Albuquerque Pereira M.d.F., Morais de Ávila L.G., Ávila Alpino G.d.C., dos Santos Cruz B.C., Almeida L.F., Macedo Simões J., Ladeira Bernardes A., Xisto Campos I., de Oliveira Barros Ribon A., de Oliveira Mendes T.A. (2023). Milk kefir alters fecal microbiota impacting gut and brain health in mice. Appl. Microbiol. Biotechnol..

[187] van de Wouw M., Walsh C.J., Vigano G.M.D., Lyte J.M., Boehme M., Gual-Grau A., Crispie F., Walsh A.M., Clarke G., Dinan T.G. (2021). Kefir ameliorates specific microbiota-gut-brain axis impairments in a mouse model relevant to autism spectrum disorder. Brain Behav. Immun..

[188] Wu Z., Wang P., Pan D., Zeng X., Guo Y., Zhao G. (2021). Effect of adzuki bean sprout fermented milk enriched in γ-aminobutyric acid on mild depression in a mouse model. J. Dairy Sci..

[189] He W., Song H., Yang Z., Zhao S., Min J., Jiang Y. (2024). Beneficial effect of GABA-rich fermented milk whey on nervous system and intestinal microenvironment of aging mice induced by D-galactose. Microbiol. Res..

[190] Han M., Dong Y., Wang S., Huang X., Bai C., Gai Z. (2024). Regulation of gut microbiota and serum neurotransmitters in mice by *Streptococcus thermophilus* GA8-and *Lacticaseibacillus rhamnosus* HAO9-fermented milk containing high levels of gamma-aminobutyric acid. J. Sci. Food Agric..

[191] Sousa R.J.M., Baptista J.A.B., Silva C.C.G. (2022). Consumption of fermented dairy products is associated with lower anxiety levels in Azorean university students. Front. Nutr..

[192] Jena R., Choudhury P.K. (2024). Lactic acid bacteria in fermented dairy foods: Gamma-aminobutyric acid (GABA) production and its therapeutic implications. Food Biosci..

[193] Qian X., Li Q., Zhu H., Chen Y., Lin G., Zhang H., Chen W., Wang G., Tian P. (2024). Bifidobacteria with indole-3-lactic acid-producing capacity exhibit psychobiotic potential via reducing neuroinflammation. Cell Rep. Med..

[194] Madison C.A., Hillbrick L., Kuempel J., Albrecht G.L., Landrock K.K., Safe S., Chapkin R.S., Eitan S. (2023). Intestinal epithelium aryl hydrocarbon receptor is involved in stress sensitivity and maintaining depressive symptoms. Behav. Brain Res..

[195] Anand S., Mande S.S. (2022). Host-microbiome interactions: Gut-Liver axis and its connection with other organs. NPJ Biofilms Microbiomes.

[196] Du Y., He C., An Y., Huang Y., Zhang H., Fu W., Wang M., Shan Z., Xie J., Yang Y. (2024). The Role of Short Chain Fatty Acids in Inflammation and Body Health. Int. J. Mol. Sci..

[197] Güler M.S., Arslan S., Ağagündüz D., Cerqua I., Pagano E., Canani R.B., Capasso R. (2025). Butyrate: A potential mediator of obesity and microbiome via different mechanisms of actions. Food Res. Int..

[198] Münte E., Hartmann P. (2025). The Role of Short-Chain Fatty Acids in Metabolic Dysfunction-Associated Steatotic Liver Disease and Other Metabolic Diseases. Biomolecules.

[199] Ano Y., Yoshino Y., Kutsukake T., Ohya R., Fukuda T., Uchida K., Takashima A., Nakayama H. (2019). Tryptophan-related dipeptides in fermented dairy products suppress microglial activation and prevent cognitive decline. Aging.

[200] Tuigunov D., Sinyavskiy Y., Nurgozhin T., Zholdassova Z., Smagul G., Omarov Y., Dolmatova O., Yeshmanova A., Omarova I. (2025). Precision Nutrition and Gut-Brain Axis Modulation in the Prevention of Neurodegenerative Diseases. Nutrients.

[201] Qin P., Berzina L., Geiker N.R.W., Sandby K., Krarup T., Kristiansen K., Magkos F. (2026). Associations Between Gut Microbiome Enterotypes and Body Weight Change During Whole Milk Consumption. Nutrients.

[202] Lee S., You H., Lee M., Kim D., Jung S., Park Y., Hyun S. (2021). Different Reactions in Each Enterotype Depending on the Intake of Probiotic Yogurt Powder. Microorganisms.

[203] Horvath T.D., Ihekweazu F.D., Haidacher S.J., Ruan W., Engevik K.A., Fultz R., Hoch K.M., Luna R.A., Oezguen N., Spinler J.K. (2022). *Bacteroides ovatus* colonization influences the abundance of intestinal short chain fatty acids and neurotransmitters. Iscience.

[204] Bah Y.R., Baba K., Mustafa D., Watanabe S., Takeda A.K., Yamashita T., Kasahara K. (2025). Bacteroides- and Prevotella-enriched gut microbial clusters associate with metabolic risks. Gut Pathog..

[205] Wacklin P., Mäkivuokko H., Alakulppi N., Nikkilä J., Tenkanen H., Räbinä J., Partanen J., Aranko K., Mättö J. (2011). Secretor genotype (*FUT2* gene) is strongly associated with the composition of *Bifidobacteria* in the human intestine. PLoS ONE.

[206] Giampaoli O., Conta G., Calvani R., Miccheli A. (2020). Can the FUT2 non-secretor phenotype associated with gut microbiota increase the children susceptibility for type 1 diabetes? A mini review. Front. Nutr..

[207] Kamitaki N., Handsaker R.E., Hujoel M.L.A., Mukamel R.E., Usher C.L., McCarroll S.A., Loh P.-R. (2026). Human and bacterial genetic variation shape oral microbiomes and health. Nature.

[208] Goodrich J.K., Waters J.L., Poole A.C., Sutter J.L., Koren O., Blekhman R., Beaumont M., Van Treuren W., Knight R., Bell J.T. (2014). Human genetics shape the gut microbiome. Cell.

[209] Han S., Lu Y., Xie J., Fei Y., Zheng G., Wang Z., Liu J., Lv L., Ling Z., Berglund B. (2021). Probiotic Gastrointestinal Transit and Colonization After Oral Administration: A Long Journey. Front. Cell. Infect. Microbiol..

[210] Hughes R.L., Kable M.E., Marco M., Keim N.L. (2019). The role of the gut microbiome in predicting response to diet and the development of precision nutrition models. Part II: Results. Adv. Nutr..

[211] Zhao F., Tie N., Kwok L.Y., Ma T., Wang J., Man D., Yuan X., Li H., Pang L., Shi H. (2024). Baseline gut microbiome as a predictive biomarker of response to probiotic adjuvant treatment in gout management. Pharmacol. Res..

[212] Rowland I., Gibson G., Heinken A., Scott K., Swann J., Thiele I., Tuohy K. (2018). Gut microbiota functions: Metabolism of nutrients and other food components. Eur. J. Nutr..

[213] Lewandowski R. (2026). The gut microbiome as a rainforest: Probiotic colonization resistance, functional effects, and next-generation strategies. FEMS Microbiol. Lett..

[214] Walter J., Maldonado-Gómez M.X., Martínez I. (2018). To engraft or not to engraft: An ecological framework for gut microbiome modulation with live microbes. Curr. Opin. Biotechnol..

[215] Zmora N., Zilberman-Schapira G., Suez J., Mor U., Dori-Bachash M., Bashiardes S., Kotler E., Zur M., Regev-Lehavi D., Brik R.B.-Z. (2018). Personalized gut mucosal colonization resistance to empiric probiotics is associated with unique host and microbiome features. Cell.

[216] Suez J., Zmora N., Zilberman-Schapira G., Mor U., Dori-Bachash M., Bashiardes S., Zur M., Regev-Lehavi D., Brik R.B.-Z., Federici S. (2018). Post-antibiotic gut mucosal microbiome reconstitution is impaired by probiotics and improved by autologous FMT. Cell.

[217] Fernández-Alonso M., Aguirre Camorlinga A., Messiah S.E., Marroquin E. (2022). Effect of adding probiotics to an antibiotic intervention on the human gut microbial diversity and composition: A systematic review. J. Med. Microbiol..

[218] Napier B.A., Allegretti J.R., Feuerstadt P., Kelly C.R., Van Hise N.W., Jäger R., Stuivenberg G.A., Kassam Z., Reid G. (2026). Multi-Species Synbiotic Supplementation After Antibiotics Promotes Recovery of Microbial Diversity and Function, and Increases Gut Barrier Integrity: A Randomized, Placebo-Controlled Trial. Antibiotics.

[219] Hoffmann Sardá F.A., Giuntini E.B., Oliveira A., Souza G.S., Prado S.B.R., Taddei C.R., Tadini C.C., Bittinger K., Bushman F.D., Menezes E.W. (2025). Baseline intestinal microbiota composition influences response to a real-world dietary fiber intervention. NPJ Biofilms Microbiomes.

[220] Zhou J., Ho V. (2023). Role of Baseline Gut Microbiota on Response to Fiber Intervention in Individuals with Irritable Bowel Syndrome. Nutrients.

[221] Procházková N., Falony G., Dragsted L.O., Licht T.R., Raes J., Roager H.M. (2023). Advancing human gut microbiota research by considering gut transit time. Gut.

[222] Le Roy C.I., Kurilshikov A., Leeming E.R., Visconti A., Bowyer R.C.E., Menni C., Falchi M., Koutnikova H., Veiga P., Zhernakova A. (2022). Yoghurt consumption is associated with changes in the composition of the human gut microbiome and metabolome. BMC Microbiol..

[223] Lisko D.J., Johnston G.P., Johnston C.G. (2017). Effects of dietary yogurt on the healthy human gastrointestinal (GI) microbiome. Microorganisms.

[224] Thriene K., Stanislas V., Huang K.D., Strowig T., Michels K.B. (2026). Impact of Yogurt and Rolled Oats Consumption on the Gut Microbiome: A Randomized Crossover Study Displaying Individual Responses and General Resilience. J. Nutr..

[225] Lu J., Zhang W., He Y., Jiang M., Liu Z., Zhang J., Zheng L., Zhou B., Luo J., He C. (2025). Multi-omics decodes host-specific and environmental microbiome interactions in sepsis. Front. Microbiol..

[226] Go D., Yeon G.-H., Park S.J., Lee Y., Koh H.G., Koo H., Kim K.H., Jin Y.-S., Sung B.H., Kim J. (2024). Integration of metabolomics and other omics: From microbes to microbiome. Appl. Microbiol. Biotechnol..

[227] Fu W., Liu Y., Li R., Jin H. (2025). Integrating Mendelian Randomization and Machine Learning to Identify Hypoxia-Related Diagnostic Biomarkers and Causal Relationship in COPD. Int. J. Chronic Obstr. Pulm. Dis..

[228] Li P., Luo H., Ji B., Nielsen J. (2022). Machine learning for data integration in human gut microbiome. Microb. Cell Fact..

[229] Armet A.M., Deehan E.C., O’sullivan A.F., Mota J.F., Field C.J., Prado C.M., Lucey A.J., Walter J. (2022). Rethinking healthy eating in light of the gut microbiome. Cell Host Microbe.

[230] Ghailan A.Z., Niamah A.K. (2025). *Streptococcus thermophilus*: Metabolic Properties, Functional Features, and Useful Applications. Appl. Microbiol..

[231] Catassi G., Mateo S.G., Occhionero A.S., Esposito C., Giorgio V., Aloi M., Gasbarrini A., Cammarota G., Ianiro G. (2024). The importance of gut microbiome in the perinatal period. Eur. J. Pediatr..

[232] Xu D., Wan F. (2025). Breastfeeding and infant gut microbiota: Influence of bioactive components. Gut Microbes.

[233] Lordan C., Roche A.K., Delsing D., Nauta A., Groeneveld A., MacSharry J., Cotter P.D., van Sinderen D. (2024). Linking human milk oligosaccharide metabolism and early life gut microbiota: Bifidobacteria and beyond. Microbiol. Mol. Biol. Rev..

[234] Inchingolo F., Inchingolo A.M., Latini G., Ferrante L., de Ruvo E., Campanelli M., Longo M., Palermo A., Inchingolo A.D., Dipalma G. (2024). Difference in the Intestinal Microbiota between Breastfeed Infants and Infants Fed with Artificial Milk: A Systematic Review. Pathogens.

[235] Odiase E., Frank D.N., Young B.E., Robertson C.E., Kofonow J.M., Davis K.N., Berman L.M., Krebs N.F., Tang M. (2023). The gut microbiota differ in exclusively breastfed and formula-fed United States infants and are associated with growth status. J. Nutr..

[236] Wickramasinghe S., Pacheco A.R., Lemay D.G., Mills D.A. (2015). Bifidobacteria grown on human milk oligosaccharides downregulate the expression of inflammation-related genes in Caco-2 cells. BMC Microbiol..

[237] Wong C.B., Huang H., Ning Y., Xiao J. (2024). Probiotics in the New Era of Human Milk Oligosaccharides (HMOs): HMO Utilization and Beneficial Effects of *Bifidobacterium longum* subsp. *infantis* M-63 on Infant Health. Microorganisms.

[238] Laursen M.F., Sakanaka M., von Burg N., Mörbe U., Andersen D., Moll J.M., Pekmez C.T., Rivollier A., Michaelsen K.F., Mølgaard C. (2021). *Bifidobacterium* species associated with breastfeeding produce aromatic lactic acids in the infant gut. Nat. Microbiol..

[239] Duranti S., Ruiz L., Lugli G.A., Tames H., Milani C., Mancabelli L., Mancino W., Longhi G., Carnevali L., Sgoifo A. (2020). *Bifidobacterium adolescentis* as a key member of the human gut microbiota in the production of GABA. Sci. Rep..

[240] Ochoa T.J., Zegarra J., Cam L., Llanos R., Pezo A., Cruz K., Zea-Vera A., Cárcamo C., Campos M., Bellomo S. (2015). Randomized controlled trial of lactoferrin for prevention of sepsis in peruvian neonates less than 2500 g. Pediatr. Infect. Dis. J..

[241] Pammi M., Preidis G.A., Tarnow-Mordi W.O. (2021). Evidence from systematic reviews of randomized trials on enteral lactoferrin supplementation in preterm neonates. Biochem. Cell Biol..

[242] Sherman M.P., Sherman J., Arcinue R., Niklas V. (2016). Randomized Control Trial of Human Recombinant Lactoferrin: A Substudy Reveals Effects on the Fecal Microbiome of Very Low Birth Weight Infants. J. Pediatr..

[243] Wernimont S., Northington R., Kullen M.J., Yao M., Bettler J. (2015). Effect of an α-lactalbumin-enriched infant formula supplemented with oligofructose on fecal microbiota, stool characteristics, and hydration status: A randomized, double-blind, controlled trial. Clin. Pediatr..

[244] Guadalupe H.-S.D., Berenice O.-R.L., Elizabeth C.-G.A., Guillermo G.-O.L., Araceli C.-O. (2025). Probiotic fermented milk and type 2 diabetes mellitus: Mechanisms, benefits, and future directions”. J. Funct. Foods.

[245] Salari A., Ghodrat S., Gheflati A., Jarahi L., Hashemi M., Afshari A. (2021). Effect of kefir beverage consumption on glycemic control: A systematic review and meta-analysis of randomized controlled clinical trials. Complement. Ther. Clin. Pract..

[246] El-Bashiti T.A., Zabut B.M., Safia F.F.A. (2019). Effect of probiotic fermented milk (Kefir) on some blood biochemical parameters among newly diagnosed type 2 diabetic adult males in Gaza governorate. Curr. Res. Nutr. Food Sci. J..

[247] Mohamadshahi M., Veissi M., Haidari F., Javid A.Z., Mohammadi F., Shirbeigi E. (2014). Effects of probiotic yogurt consumption on lipid profile in type 2 diabetic patients: A randomized controlled clinical trial. J. Res. Med. Sci. Off. J. Isfahan Univ. Med. Sci..

[248] Bellikci-Koyu E., Sarer-Yurekli B.P., Karagozlu C., Aydin-Kose F., Ozgen A.G., Buyuktuncer Z. (2022). Probiotic kefir consumption improves serum apolipoprotein A1 levels in metabolic syndrome patients: A randomized controlled clinical trial. Nutr. Res..

[249] Morales G., Bugueño C., Valenzuela R., Chamorro R., Leiva C., Gotteland M., Trunce-Morales S., Pizarro-Aranguiz N., Durán-Agüero S. (2025). Association between cheese consumption but not other dairy products and lower obesity risk in adults. PLoS ONE.

[250] D’Amico F., Decembrino N., Muratore E., Turroni S., Muggeo P., Mura R., Perruccio K., Vitale V., Zecca M., Prete A. (2022). Oral Lactoferrin Supplementation during Induction Chemotherapy Promotes Gut Microbiome Eubiosis in Pediatric Patients with Hematologic Malignancies. Pharmaceutics.

[251] Sortino O., Hullsiek K.H., Richards E., Rupert A., Schminke A., Tetekpor N., Quinones M., Prosser R., Schacker T., Sereti I. (2019). The Effects of Recombinant Human Lactoferrin on Immune Activation and the Intestinal Microbiome Among Persons Living with Human Immunodeficiency Virus and Receiving Antiretroviral Therapy. J. Infect. Dis..

[252] Ruiz-Rico M., Ye H., O’Callaghan T.F., O’Toole P.W., McCarthy E.K. (2025). Iron-saturated bovine lactoferrin preserves microbiota diversity and healthy ageing-associated taxa in an in vitro colon model of elderly gut microbiota (Iron-saturated bovine lactoferrin impact on elderly gut microbiota). PLoS ONE.

[253] Gyriki D., Nikolaidis C.G., Bezirtzoglou E., Voidarou C., Stavropoulou E., Tsigalou C. (2025). The gut microbiota and aging: Interactions, implications, and interventions. Front. Aging.

[254] Gao W., Lee H.Y., Min K.J. (2026). Aging and the microbiome: Implications for health and disease. BMB Rep..

[255] Konstanti P., van Splunter M., van den Brink E., Belzer C., Nauta A., van Neerven R.J.J., Smidt H. (2022). The Effect of Nutritional Intervention with Lactoferrin, Galactooligosacharides and Vitamin D on the Gut Microbiota Composition of Healthy Elderly Women. Nutrients.

[256] Elison E., Vigsnaes L.K., Rindom Krogsgaard L., Rasmussen J., Sørensen N., McConnell B., Hennet T., Sommer M.O., Bytzer P. (2016). Oral supplementation of healthy adults with 2′-O-fucosyllactose and lacto-N-neotetraose is well tolerated and shifts the intestinal microbiota. Br. J. Nutr..

[257] Rein M.J., Renouf M., Cruz-Hernandez C., Actis-Goretta L., Thakkar S.K., da Silva Pinto M. (2013). Bioavailability of bioactive food compounds: A challenging journey to bioefficacy. Br. J. Clin. Pharmacol..

[258] Nagpal R., Behare P., Rana R., Kumar A., Kumar M., Arora S., Morotta F., Jain S., Yadav H. (2011). Bioactive peptides derived from milk proteins and their health beneficial potentials: An update. Food Funct..

[259] Chen J., Zhang T., Ashaolu T.J., Zhao C. (2025). Invited review: Structural-functional synergies of lactoferrin-bioactive compound complexes: Multidisciplinary applications. J. Dairy Sci..

[260] Valle Vargas M.F., Ruiz Pardo R.Y., Villamil-Díaz L., Alean J., Santagapita P.R., Quintanilla-Carvajal M.X. (2025). Encapsulation improves viability and stability of spray-dried Lactococcus lactis A12 for inclusion in fish feed. PLoS ONE.

[261] Golowczyc M.A., Silva J., Abraham A.G., De Antoni G.L., Teixeira P. (2010). Preservation of probiotic strains isolated from kefir by spray drying. Lett. Appl. Microbiol..

[262] Fijałkowski P., Pomastowski P., van Eldik R., Rafińska K. (2025). Multifunctional role of Lactoferrin in metal ion interactions and biomedical applications: A review. Int. J. Biol. Macromol..

[263] Saleem G.N., Gu R., Qu H., Bahar Khaskheli G., Rashid Rajput I., Qasim M., Chen X. (2024). Therapeutic potential of popular fermented dairy products and its benefits on human health. Front. Nutr..

[264] Manca C., Boubertakh B., Leblanc N., Deschênes T., Lacroix S., Martin C., Houde A., Veilleux A., Flamand N., Muccioli G.G. (2020). Germ-free mice exhibit profound gut microbiota-dependent alterations of intestinal endocannabinoidome signaling. J. Lipid Res..

[265] Plaza-Díaz J., Fontana L., Gil A. (2018). Human Milk Oligosaccharides and Immune System Development. Nutrients.

[266] Akdemir Evrendilek G. (2026). Designing Functional Foods Beyond Bioactivity: Integrating Processing, Safety, and Regulatory Readiness. Appl. Sci..

[267] Shan F., Liu L., Li L., Wang W., Bi Y., Li M. (2025). Management, Safety, and Efficacy Evaluation of Nutraceutical and Functional Food: A Global Perspective. Compr. Rev. Food Sci. Food Saf..

[268] Ray P.R., Manik S., Sen C. (2026). Current policies and regulations on food proteins and bioactive peptides. Non-Bovine Milk Derived Bioactive Peptides.

[269] Farnworth E.R. (2008). The evidence to support health claims for probiotics. J. Nutr..

